# Efficacy and safety of combined low‐dose rituximab regimen for chronic inflammatory demyelinating polyradiculoneuropathy

**DOI:** 10.1002/acn3.52270

**Published:** 2024-12-11

**Authors:** Ying Du, Qi Yan, Chuan Li, Wenping Zhu, Chao Zhao, Yunfeng Hao, Lin Li, Dan Yao, Xuan Zhou, Ying Li, Yuting Dang, Rong Zhang, Lin Han, Yuanyuan Wang, Tao Hou, Juan Li, Hailin Li, Panpan Jiang, Pei Wang, Fenying Chen, Tingge Zhu, Juntong Liu, Shuyu Liu, Lan Gao, Yingjun Zhao, Wei Zhang

**Affiliations:** ^1^ Department of Neurology, Tangdu Hospital Fourth Military Medical University Xi'an 710038 Shaanxi China; ^2^ Xi'an Medical University Xi'an 710021 Shaanxi China; ^3^ Department of Internal Medicine Qianxian Traditional Chinese Medicine Hospital Xianyang 713300 Shaanxi China; ^4^ Department of Neurology Fuping County Hospital Weinan 711700 Shaanxi China; ^5^ Department of Neurology Lantian Country People's Hospital Xi'an 710500 Shaanxi China; ^6^ Department of Neurology Pingli County Hospital Ankang 725500 Shaanxi China; ^7^ Department of Neurology The Second Hospital of Weinan Weinan 711700 Shaanxi China; ^8^ Department of Internal Medicine Baishui County Hospital Weinan 715600 Shaanxi China; ^9^ Department of Neurology and Department of Neuroscience, the First Affiliated Hospital of Xiamen University, Institute of Neuroscience, Fujian Provincial Key Laboratory of Neurodegenerative Disease and Aging Research, School of Medicine Xiamen University Xiamen 361005 Fujian China

## Abstract

**Objective:**

To determine the efficacy and safety of combined low‐dose rituximab with conventional therapy for chronic inflammatory demyelinating polyradiculoneuropathy (CIDP) treatment.

**Methods:**

Total 73 patients with CIDP were enrolled for the retrospective cohort study, and divided into conventional first‐line therapy cohort (*n* = 40) and combined low‐dose rituximab (100 mg per infusion) cohort (*n* = 33). The outcome measures include scores of I‐RODS, mRS, INCAT, ONLS, TSS, and COMPASS 31 scale at baseline and regular four visits (4, 16, 28, and 52 weeks), as well as proportion of favorable response and outcome, corticosteroids dosage, and deterioration occurrence during follow‐up.

**Results:**

Compared to conventional therapy cohort, combined rituximab cohort presented better improvements and higher proportion of favorable response in scales assessments at each visit, as well as significantly reduced corticosteroids dosage and deterioration occurrence during the follow‐up. Analyses of subgroups showed better improvements in both typical CIDP and CIDP variants in combined rituximab cohort than those in conventional therapy cohort, but had no differences between each other. Early initiating combined rituximab regimen (<10 weeks) showed better improvements than delayed initiation (≥10 weeks) at the first three visits within 28 weeks, while had no difference in favorable prognoses at the last visit of 52 weeks after once reinfusion. No rituximab correlated serious adverse events were reported in our patients.

**Interpretation:**

Our simplified regimen of combined low‐dose rituximab has been firstly demonstrated for the better efficacy and safety than conventional therapy in CIDP treatment.

## Introduction

Chronic inflammatory demyelinating polyneuropathy (CIDP) is an acquired and heterogeneous polyneuropathy with autoimmune‐mediated pathogenesis, which can be diagnosed by guidelines from the European Academy of Neurology/Peripheral Nerve Society (EAN/PNS)^1^. Patients usually experience progressive deterioration in muscle weakness and/or sensory impairment of limbs with reduced tendon reflexes over at least 8 weeks, and divided into typical CIDP and CIDP variants (including distal, multifocal, focal, motor, or sensory CIDP) according to diagnosis criteria^2^. The demyelination and axonal damage of peripheral nerves from an aberrant immune response, can lead to a poor disability with motor dysfunction, sensory, and/or autonomic symptom^3^.

The therapeutic regimen of CIDP usually consisted of induction and maintenance treatment. Oral or intravenous high‐dose corticosteroids, intravenous immunoglobulin (IVIG), and plasma exchange (PLEX) were recommended as first‐line options for induction treatment, then followed by steroid‐sparing or subcutaneous immunoglobulin (SCIg) with a gradual taper to the lowest effective dosage for maintenance^4^, ^5^, ^6^. A RCT study showed that treatment of CIDP with IVIg for 6 months was less frequently discontinued because of inefficacy, adverse events, or intolerance than the treatment with intravenous methylprednisolone. However, more patients on IVIg worsened and required further therapy than did those on methylprednisolone after therapy discontinuation^7^. IVIg produced short‐term improvement for 2–6 weeks than placebo in this study, while another recent study showed the benefit of IVIg compared with placebo in terms of improved disability score persisted for 24 weeks^8^, ^9^. PLEX also provided short‐term improvement, but potentially rapid deterioration might occur afterward^10^. Switching to weekly SCIg for maintenance, the dose was tailored, but still almost one‐third patients had a deterioration or were withdrawn during a limited study period of 24 weeks^11^. Given the deficiency in current treatments, a more aggressive and precisive immunotherapy should be crucial for CIDP during both induction and maintenance stage.

Although the exact pathogenic mechanism of CIDP remained unclear, the role of B‐cell mediated humoral immunity received significant attention^12^. Recently, rituximab, an antibody specifically targeting B‐cell depletion initially for lymphoma treatment, has gradually presented potential advantages as an off‐label attempt in treating CIDP or other autoimmune neuropathies caused by nodal–paranodal neurofascin (NF), contactin 1 (CNTN1), and contactin‐associated protein 1 (Caspr1) antibodies^13^, ^14^, ^15^, ^16^. The high‐dose regimens of rituximab for CIDP were empirically derived from the protocols against high tumor burden, including 375 mg/m^2^ weekly for four consecutive weeks or two doses of 1000 mg 2 weeks apart for induction treatment, then followed by high‐dose reinfusions at fixed intervals for immunosuppressive maintenance, because of circulating B‐cell below the detectable range for 6–8 months after infusion^17^, ^18^, ^19^.

Recently, we and other researchers have practiced the low‐dose rituximab regimen in a series of neuroimmune diseases in retrospective study, such as neuromyelitis optica (NMOSD)^20^, myasthenia gravis (MG)^21^, multiple sclerosis (MS)^22^, neuro‐Behçet's disease (NBD),^23^ and autoimmune encephalitis (AE)^24^, which showed sufficient depletion of B cells and favorable clinical outcomes with reduced side‐effect and cost. The regimen was an induction of 100 mg rituximab once a week for three cycles, followed by reinfusions 100 mg once at regular intervals of 28 weeks for maintenance, and the total dosage was only 400 mg. In the present study, by comparing with conventional first‐line therapy, we explored the efficacy and safety of combined low‐dose rituximab for CIDP treatment.

## Methods

### Standard protocol approvals

This study was performed according to the Declaration of Helsinki, and approved by the Ethical Committee of Tangdu Hospital, Fourth Military Medical University. Moreover, we have provided patients and their relatives detailed information about the disease, and obtained the consent of the patients or their legal representatives to conventional first‐line therapy and low‐dose rituximab (100 mg per infusion) treatment, while written informed consent was obtained from all patients or their legal representatives.

### Study population

The study retrospectively collected and analyzed 73 Chinese patients with CIDP diagnosed in the department of neurology from seven tertiary hospitals between March 2018 and March 2023. Among them, 33 patients in combined rituximab cohort met the following inclusion criteria: (1) patients (≥18 years) with diagnosis of CIDP according to published criteria from the guidelines of EAN/PNS^1^; (2) the Inflammatory Rasch‐built Overall Disability Scale (I‐RODS) ≤80 centile scores or the modified Rankin Scale (mRS) ≥2 scores at baseline screening; (3) any documented treatment with low‐dose rituximab (100 mg per infusion), and available information on the number and timing of infusions; (4) before combined rituximab initiation, patients received prior first‐line immunotherapy including oral or intravenous corticosteroids, and/or IVIG 2 g/kg over 5 days (0.4 g/kg/day), followed by oral corticosteroids taper‐off. In addition, according to the consistent inclusion criteria (except rituximab treatment), 40 patients with only conventional therapy for CIDP were enrolled as a control cohort.

The exclusion criteria were as follows: (1) autoimmune neuropathy caused by the known pathogenic antibodies to nodal‐paranodal antigens, such as NF140/155/186, CNTN1, and Caspr1; (2) neuropathy with monoclonal gammopathies, such as IgG or IgA monoclonal gammopathy of undetermined significance (MGUS), anti‐myelin‐associated glycoprotein (MAG) IgM antibodies, IgM monoclonal gammopathy without antibodies to MAG, polyneuropathy‐organomegaly‐endocrinopathy‐M‐protein‐skin (POEMS) syndrome, multiple myeloma, or amyloid light‐chain (AL) amyloidosis; (3) neuropathy with paraneoplastic antibodies, such as anti‐amphiphysin, anti‐Hu, anti‐CV2, anti‐Yo antibody, and so on; (4) disease complicated by acute or chronic viral or bacterial infections, such as HIV, latent hepatitis B, tuberculosis, syphilis, viral encephalitis, and so on; and (5) presence of other severe systemic or neurological complications, such as tumor, connective tissue diseases, stroke, myasthenia gravis, and so on.

### Study design

**Figure 1:**
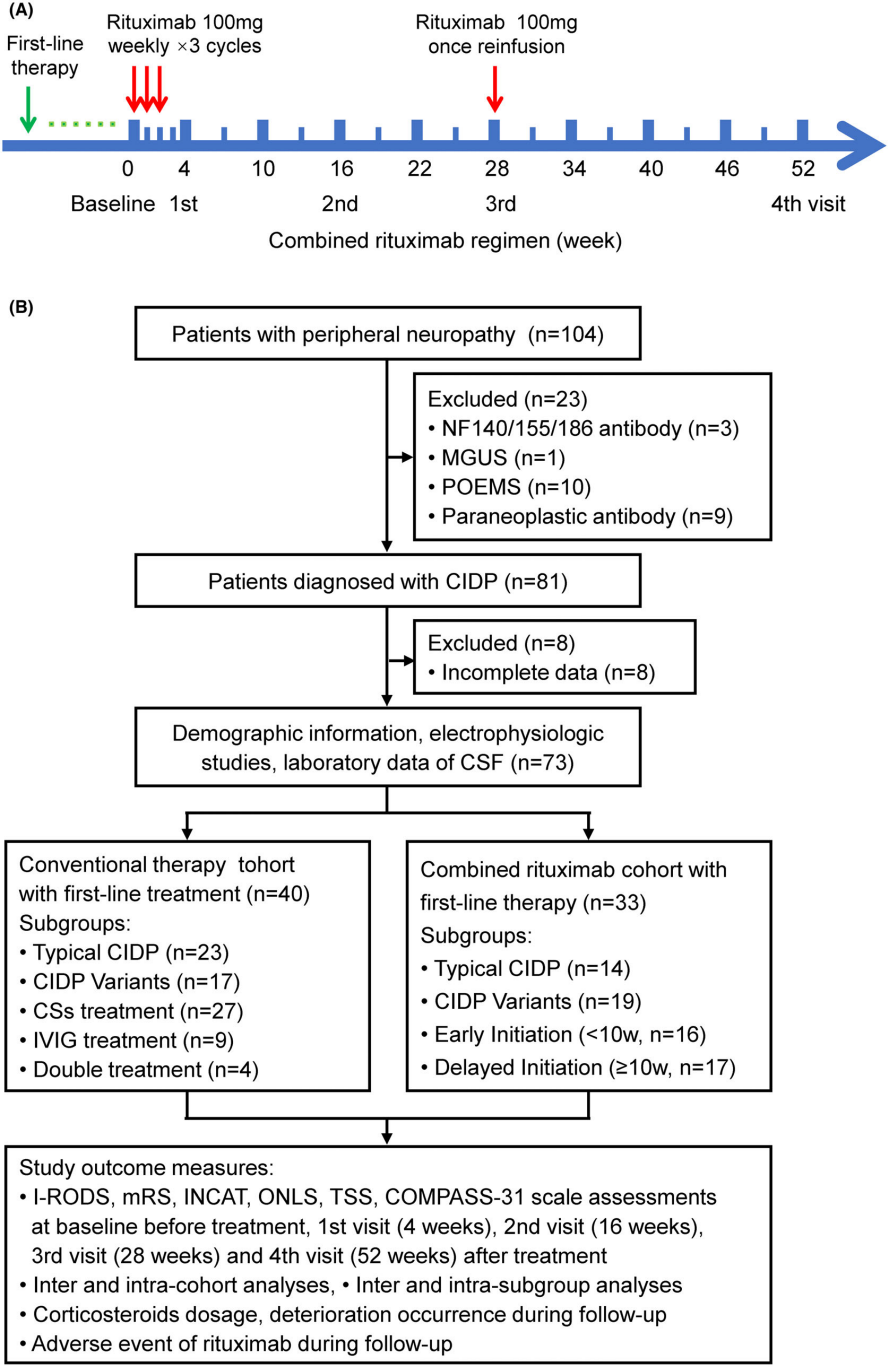
Flow chart of study design. CIDP, chronic inflammatory demyelinating polyradiculoneuropathy; COMPASS, Composite Autonomic Symptom Score; CSF, cerebrospinal fluid; INCAT, Inflammatory Neuropathy Cause and Treatment disability scale; I‐RODS, Inflammatory Rasch‐built Overall Disability Scale; MGUS, monoclonal gammopathy of undetermined significance; mRS, modified Rankin Scale; NF, neurofascin; ONLS, Overall Neuropathy Limitation Scale; POEMS, polyneuropathy‐organomegaly‐endocrinopathy‐M‐protein‐skin syndrome; TSS, Total Symptoms Score.

The 73 patients with CIDP were divided into control cohort with conventional therapy (*n* = 40) and combined rituximab cohort (*n* = 33). The regimen of combined rituximab was an induction of 100 mg once a week for three cycles, followed by reinfusions 100 mg once at regular intervals of 28 weeks for maintenance, and the total dosage was only 400 mg (Fig. 1A). Simultaneously, detailed clinical assessments by a series of scales for CIDP were performed in both cohorts at baseline and regular four visits (4, 16, 28, and 52 weeks), such as I‐RODS and mRS for evaluating disability, Inflammatory Neuropathy Cause and Treatment (INCAT) disability scale and Overall Neuropathy Limitation Scale (ONLS) for motor dysfunctions, and Total Symptoms Score (TSS) for sensory symptoms and Composite Autonomic Symptom Score (COMPASS 31) for autonomic symptoms^25^, ^26^, ^27^, ^28^, ^29^, ^30^ (Table S1). Data on any immunotherapy and side effects of rituximab were recorded (Fig. 1B).

### Study outcomes

The primary efficacy outcome was, compared to control cohort with conventional therapy for CIDP, the significant improvements of I‐RODS scores at each visit in combined rituximab cohort. The secondary outcome measures were better improvements in mRS, INCAT, ONLS, TSS, and COMPASS 31 scores at each visit than control cohort, as well as significantly reduced corticosteroids dosage (normalization as prednisone) and deterioration occurrence during the follow‐up.

As described in EAN/PNS, the changes required to define response have not been adequately validated so far, and higher values stand for stricter specificity of improvement.^1^ Thus, we set the standard of “favorable response” and “favorable outcome” to strictly assessing efficacy of combined low‐dose rituximab. To adjust the possible differences in baseline status, the parameter of “favorable response” was analyzed inter‐cohort at each visit under the following definition: improvement of I‐RODS ≥10 centile scores or achievement ≥80 centile scores, improvement of mRS ≥1 score or achievement ≤1 score, improvement of INCAT ≥2 score or achievement ≤1 score, improvement of ONLS ≥2 score or achievement ≤1 score, and improvement of TSS ≥1.66 scores or achievement ≤1.66 scores. The deterioration occurrence was also analyzed inter‐cohort under the following definition of deterioration: from the second visit (16 weeks), decreased I‐RODS centile scores ≥10% of baseline, increased mRS scores ≥1 score from baseline, increased INCAT scores ≥1 score from baseline, increased ONLS scores ≥2 scores from baseline, or increased TSS scores ≥1.66 scores from baseline. Longitudinal intra‐cohort analyses of “favorable outcome” were performed at each visit after treatments under the following definition: I‐RODS ≥80 centile scores, mRS ≤1 score, INCAT ≤1 score, ONLS ≤1 score, or TSS ≤1.66 scores.

### Assistant examinations

Electrophysiologic studies were completed at baseline by two neurologists with well‐trained experience (T Zhu and F Chen) who were blinded to the clinical diagnosis, and EFNS/PNS electrodiagnostic criteria for demyelination were applied to determine the conduction abnormalities^1^. Measures included motor conduction velocity (MCV), compound muscle action potential (CMAP) amplitude, sensory conduction velocity (SCV), and sensory nerve action potential (SNAP) amplitude. Cerebrospinal fluid (CSF) analyses were performed at baseline to support the diagnosis of CIDP, and exclude other diagnoses such as malignancy or infection. (Supplemental Method).

Moreover, all patients were screened serum for monoclonal proteins or antibodies with protein electrophoresis, immunofixation, enzyme‐linked immunosorbent assay (ELISA), and cell‐based assay (CBA) as recommended by EFNS/PNS criteria for immunological testing^1^. Specifically, detected indexes included IgG, IgA or IgM monoclonal paraprotein, anti‐MAG antibody, vascular endothelial growth factor (VEGF), antibodies to nodal–paranodal antigens (anti‐NF140/155/186, anti‐CNTN1, and anti‐Caspr1 antibody), and paraneoplastic antibodies (anti‐amphiphysin, anti‐Hu, anti‐CV2, and anti‐Yo antibody). The seropositive subjects were excluded from the study.

### Statistical analyses

Continuous variables with normal distribution are presented as mean and standard deviation, and comparisons are made using independent samples t‐test or one‐way analysis of variance. Variables with non‐normal distribution are expressed as median (interquartile range), and comparisons are made using non‐parametric Mann–Whitney U or Kruskal–Wallis H tests. Categorical variables are represented as counts (percentages) and compared using chi‐square tests. Kaplan–Meier curves are employed to depict deterioration occurrence, and the log‐rank test is utilized to compare disease progression across conventional therapy and combined rituximab cohort. Differences with a two‐tailed *p*‐value <0.05 are considered statistically significant. Statistical tests were performed using GraphPad Prism9.0 (GraphPad Software, Inc., San Diego, CA) and SPSS version 26.0 (IBM, Armonk, NY, USA). (Supplemental Method).

## Results

### Patient characteristics

According to the designed inclusion and exclusion criteria, we finally identified total 73 patients with CIDP, including 40 patients in control cohort with only conventional first‐line therapy, and 33 patients in combined rituximab cohort. The gender, age, comorbidities, diagnosis and follow‐up duration, CIDP subtypes, proportion of first‐line drugs, scores in a series of scales showed no difference between two cohorts at baseline, except for higher scores in TSS for severity in combined rituximab cohort (Table 1). The data of electrophysiologic studies including the number of peripheral nerve injuries, MCV, CMAP, SCV, and SNAP (Table S2), as well as the laboratory data of blood and CSF, also showed no difference between two cohorts at baseline (Table S3).

**Table 1:** Characteristics of patient in conventional therapy and combined rituximab cohort.

	CT cohort (*n* = 40)	CR cohort (*n* = 33)	*p* value
Age, mean (SEM), years	54 (1.96)	53 (2.59)	0.902
BMI, mean (SEM), kg/m^2^	22.2 (0.65)	21.9 (0.58)	0.904
Female sex, No. (%)	16 (40)	14 (42.4)	0.511
Diagnosis duration, median (IQR), days	168 (423)	129 (292)	0.937
Follow‐up duration, mean (95% Cl), days	371 (5.25)	374 (6)	0.847
Hypertension, No. (%)	14 (35)	5 (15.2)	0.065
Cardiopathy, No. (%)	3 (7.5)	4 (12.1)	0.694
Diabetes, No. (%)	6 (15)	8 (24.2)	0.379
CIDP subtype			0.295
Typical CIDP	23 (57.5)	14 (42.4)	
CIDP variants	17 (42.5)	19 (57.6)	
Scale, score median (IQR)			
I‐RODS	55 (20)	52 (19)	0.894
mRS	3 (2)	3 (2)	0.104
INCAT	4 (2)	4 (2)	0.965
ONLS	5 (2)	5 (2)	0.787
TSS	5.6 (4.07)	7.32 (3.5)	*0.002*
COMPASS‐31	9 (11)	9 (13)	0.421
Proportion of first‐line drugs (%)			0.186
CSs	31 (70.5)	24 (54.5)	
IVIG	13 (29.5)	20 (45.5)	

BMI, body mass index (calculated as weight in kilograms divided by height in meters squared); COMPASS‐31, Composite Autonomic Symptom Score‐31; CR cohort, combined rituximab cohort; CSs, corticosteroids; CT cohort, conventional therapy cohort; INCAT, Inflammatory Neuropathy Cause and Treatment disability scale; IVIG, intravenous immunoglobulin; I‐RODS, Inflammatory Rasch‐Overall Disability Scale; mRS, modified Rankin Scale; ONLS, Overall Neuropathy Limitation Scale; SEM, standard error of the mean; TSS, Total Symptom Score.

### Comparisons of outcomes between two cohorts

**Figure 2:**
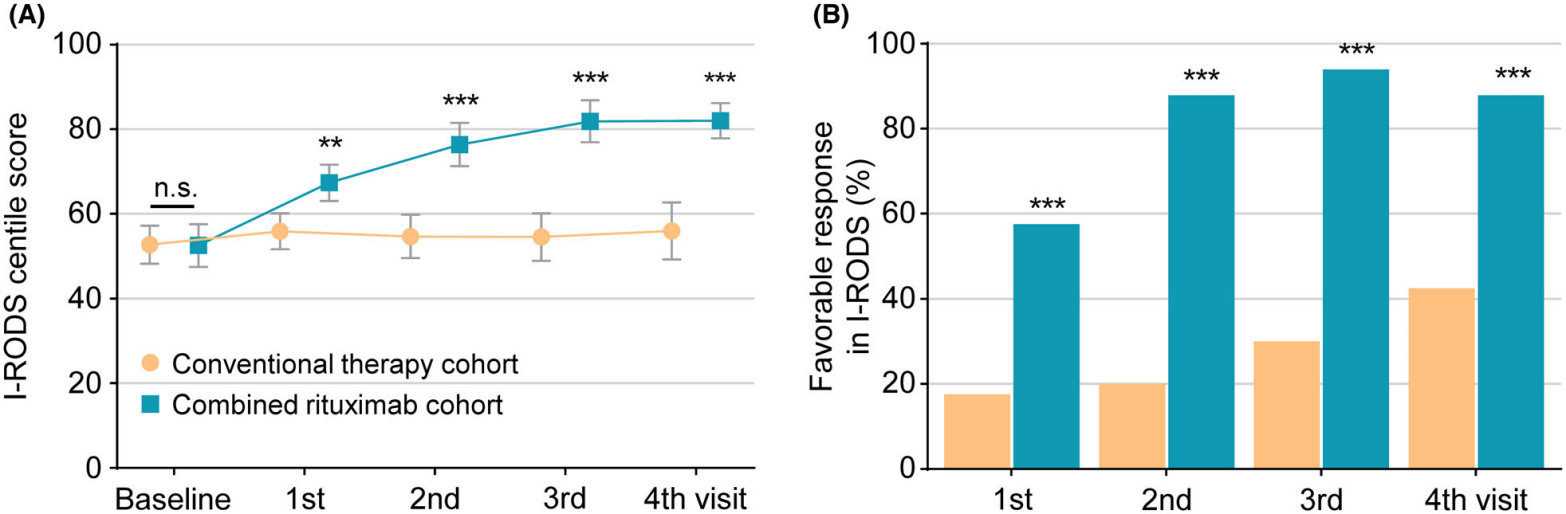
Comparisons of outcomes between conventional therapy and combined rituximab cohort. During the follow‐up of 52 weeks, combined rituximab cohort showed better improvements (A) and higher proportions of favorable response (B) in I‐RODS centile scores at each visit than those in conventional therapy cohort. **p < 0.01, ***p < 0.001.

**Figure 3:**
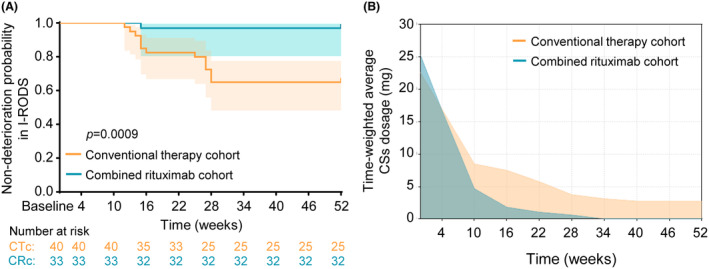
Comparisons of deterioration occurrence and corticosteroids dosage between conventional therapy and combined rituximab cohort. During the follow‐up of 52 weeks, combined rituximab cohort showed higher non‐deterioration probability (A) in I‐RODS centile scores, and lower time‐weighted average corticosteroids dosage (normalization as prednisone) (B) than those in conventional therapy cohort.

Compared to control cohort with conventional therapy for CIDP, patients in combined rituximab cohort presented no difference in I‐RODS, mRS, INCAT, ONLS, and COMPASS 31 scores at baseline, but higher scores in TSS for severity. During the follow‐up of 52 weeks, combined rituximab cohort showed better improvements and outcomes in I‐RODS centile scores at each visit than control cohort (Fig. 2A and Table 2), indicating the achievement of primary efficacy outcome as designed in our study. The similar results were also presented in mRS, INCAT, and ONLS scores, except for continuous improvements in COMPASS 31 and TSS scores just from the second visit (16 weeks), suggesting the achievement of secondary outcomes (Table 2 and Fig. S1). Meanwhile, patients in combined rituximab cohort presented higher proportions of “favorable response” (Fig. 2B) and non‐deterioration probability (Fig. 3A) in I‐RODS centile scores than those in control cohort, and the similar results were also revealed in other scales assessments (Figs. S2 and S3). The time‐weighted average corticosteroids dosage was also significantly reduced in combined rituximab cohort during the follow‐up (Fig. 3B). Altogether, these results demonstrated the better comprehensive efficacy with combined low‐dose rituximab for CIDP treatment.

**Table 2:** Comparison of clinical outcomes between conventional therapy cohort and combined rituximab cohort.

Evaluation scale, median (IQR)	Baseline			First visit			Second visit			Third visit			4th visit		
	CT cohort (*n* = 40)	CR cohort (*n* = 33)	*p* value	CT cohort (*n* = 40)	CR cohort (*n* = 33)	*p* value	CT cohort (*n* = 40)	CR cohort (*n* = 33)	*p* value	CT cohort (*n* = 40)	CR cohort (*n* = 33)	*p* value	CT cohort (*n* = 40)	CR cohort (*n* = 33)	*p* value
I‐RODS^a^															
Scores	55 (20)	52 (19)	0.894	60 (19)	65 (15.5)	0.002	56 (21.5)	76 (20)	<0.001	55 (24.5)	88 (15)	<0.001	56 (35.5)	88 (16)	<0.001
Δ Scores^g^	NA	NA	NA	0 (5.8)	12 (22)	<0.001	0 (12)	21 (15)	<0.001	0 (22.75)	28 (18)	<0.001	5 (33)	28 (22.5)	<0.001
mRS^b^															
Scores	3 (1.7)	3 (2)	0.104	3 (1)	2 (1)	0.028	3 (1.7)	1 (1)	<0.001	3 (2)	1 (1)	<0.001	3 (2)	1 (1)	<0.001
Δ Scores^g^	NA	NA	NA	0 (0)	−1 (2)	<0.001	0 (1)	−2 (1.5)	<0.001	0 (2)	−2 (2)	<0.001	0 (1.75)	−2 (2)	<0.001
INCAT^c^															
Scores	4 (2)	4 (2)	0.790	3 (2)	2 (1.5)	<0.001	3 (2)	2 (1)	*<0.001*	3 (3)	1 (1)	<0.001	3 (4)	1 (1)	<0.001
Δ Scores^g^	NA	NA	NA	0 (4)	−1 (2.5)	<0.001	0 (1)	−2 (2)	<0.001	0 (2.7)	−3 (2.5)	<0.001	−1 (3)	−3 (2.5)	<0.001
ONLS^d^															
Scores	5 (2)	5 (2)	0.787	4 (2)	3 (2)	<0.001	4 (2.75)	2 (2)	<0.001	4 (3)	1 (1)	<0.001	4 (4)	1 (1)	<0.001
Δ Scores^g^	NA	NA	NA	0 (1)	2 (2)	<0.001	0 (1.75)	−2 (2)	<0.001	0 (3.75)	−3 (1.5)	<0.001	−1 (4)	−3 (2)	<0.001
TSS^e^															
Scores	5.6 (4.2)	7.32 (3.6)	0.001	5.32 (3)	5.32 (4.1)	0.951	5.32 (4.2)	2.99 (3.1)	0.015	5.3 (4.3)	1.66 (1.8)	<0.001	4.82 (5.3)	1.33 (2.1)	0.003
Δ Scores^g^	NA	NA	NA	0 (1.2)	−2 (3.3)	<0.001	0 (1)	−4 (3)	<0.001	0 (2)	−5.3 (3.3)	<0.001	−1 (2.3)	−5.6 (3.3)	<0.001
COMPASS‐31^f^															
Scores	9.5 (10.5)	9 (13.5)	0.421	10 (10)	7 (13.5)	0.301	13.5 (13.7)	6 (7.5)	<0.001	11.5 (16)	4 (7.5)	<0.001	12 (14.2)	3 (6)	<0.001
Δ Scores^g^	NA	NA	NA	0 (0)	−2 (6.5)	<0.001	2.5 (7.7)	−5 (10)	<0.001	3 (8.7)	−6 (8.5)	<0.001	3 (12.5)	−7 (9)	<0.001

COMPASS‐31, Composite Autonomic Symptom Score‐31; CR cohort, combined rituximab cohort; CT cohort, conventional therapy cohort; INCAT, Inflammatory Neuropathy Cause and Treatment disability scale; I‐RODS, Inflammatory Rasch‐Overall Disability Scale; mRS, modified Rankin Scale; ONLS, Overall Neuropathy Limitation Scale; TSS, Total Symptom Score.

^a^ Scores on the I‐RODS ranged from 0 to 100 centile points, with lower scores indicating a more severe level of disability.

^b^ Scores on the mRS ranged from 0 to 6, with from perfect health without symptoms to death.

^c^ Scores on the INCAT ranged from 0 to 10 points, with higher scores indicating a more severe level of limb dysfunction.

^d^ Scores on the ONLS ranged from 0 to 12 points, with higher scores indicating lower levels of activities measure.

^e^ Scores on the TSS ranged from 0 to 14.64 points, with higher scores indicating a more severe level of global sensory dysfunction.

^f^ Scores on the COMPASS‐31 ranged from 0 to 75 points, with higher scores indicating a more severe level of autonomic symptoms dysfunction.

^g^ Change in scores from baseline to different visits.

### Analyses of improvements intra‐cohort

**Figure 4:**
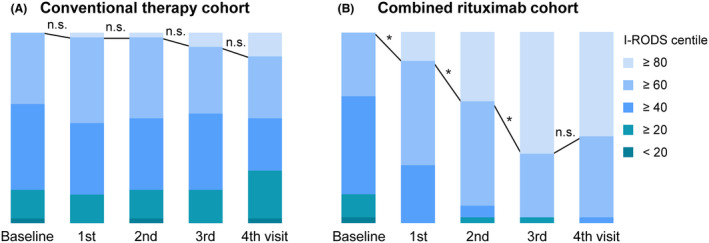
Longitudinal intra‐cohort analyses of favorable outcome in conventional therapy and combined rituximab cohort. During the follow‐up, proportions of favorable outcome in I‐RODS centile scores were not different at each visit in conventional therapy cohort (A), while the proportions were significantly increased at the first three visits within 28 weeks in combined rituximab cohort, and stably sustained till the last visit of 52 weeks (B). *p < 0.05.

Further longitudinal intra‐cohort analyses of “favorable outcome” were performed with I‐RODS, mRS, INCAT, ONLS, and TSS scale respectively. In conventional therapy cohort, proportions of “favorable outcome” in a series of scales (Fig. 4A and Fig. S4), as well as changes between baseline and each visit (Table S4), presented no difference during the follow‐up. While in combined rituximab cohort, scores in a series of scales were significantly improved at each visit, except for a continuous improvement after the second visit in COMPASS 31 scale (Table S4). Meanwhile, proportions of “favorable outcome” were also continuously and significantly increased during the first three visits (within 28 weeks) after induction treatment with low‐dose rituximab, and then stably sustained for at least 24 weeks till the end of study (52 weeks) by maintenance with once rituximab refusion at the 3rd visit (28 weeks; Fig. 4B and Fig. S4). These results indicated both short‐ and long‐term favorable prognoses of CIDP by induction and maintenance treatment with combined low‐dose rituximab.

### Analyses for subgroups

**Figure 5:**
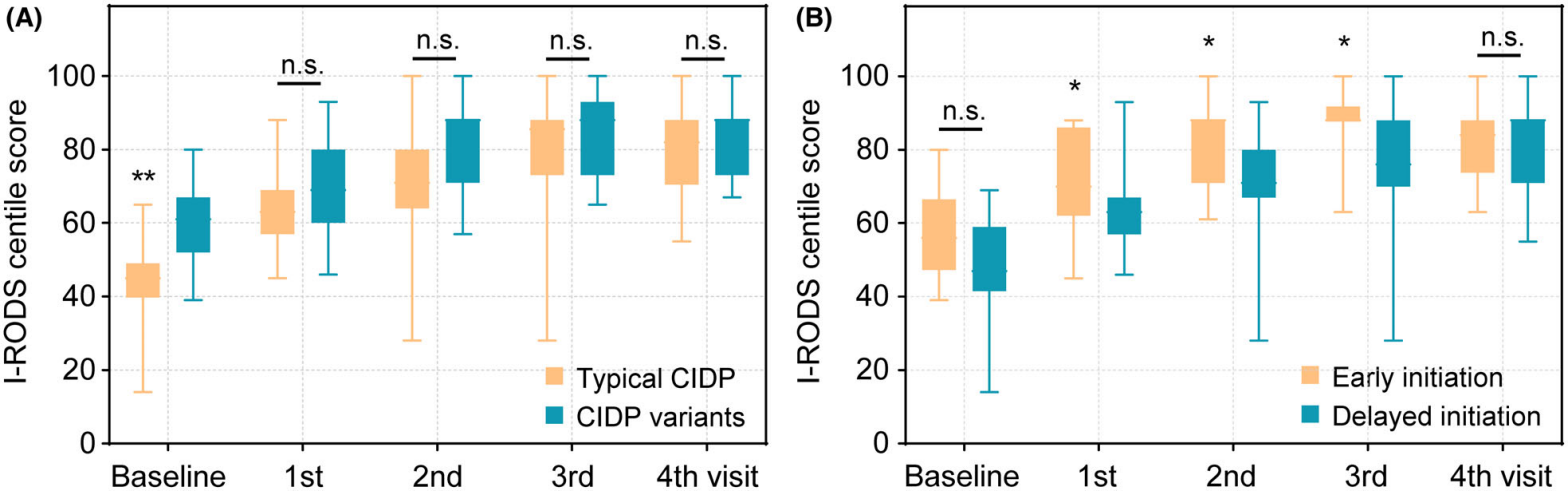
Analyses of subgroups in combined rituximab cohort. During the follow‐up, the favorable improvements of I‐RODS centile scores were no difference at each visit between the typical CIDP and CIDP variants subgroup in combined rituximab cohort (A). Early initiating combined rituximab regimen (<10 weeks) showed better improvements of I‐RODS centile scores than delayed initiation subgroup (≥10 weeks) at the first three visits within 28 weeks, while had no difference in favorable prognosis at the last visit of 52 weeks (B). *p < 0.05, **p < 0.01.

In the conventional therapy cohort, there was no difference in outcome between different first‐line drugs, as well as different subtypes of CIDP (Tables S5–S8). The combined rituximab cohort showed better outcomes than conventional therapy in typical CIDP and CIDP variants subgroup, but had no significant differences between each other (Fig. 5A; Tables S9 and S10; Fig. S5), indicating the favorable efficacy of combined low‐dose rituximab for both CIDP subtypes. Moreover, analyses for opportunity of initiating combined rituximab regimen showed better improvements in early initiation (<10 weeks) subgroup than those in delayed initiation (≥10 weeks) during the first three visits within 28 weeks, but not at the last visit of 52 weeks (Fig. 5B; Tables S12 and S13; Fig. S6), suggesting that early initiation of combined rituximab was beneficial for rapid improvements in a short‐term but no influence on the favorable long‐term prognoses of CIDP.

### Adverse effects and safety of rituximab

In total 33 patients with combined low‐dose rituximab, 4 had infusion‐related symptom which presented as skin rash or pruritus during the administration of rituximab. However, the symptoms gradually disappeared after oral cetirizine. No rituximab correlated serious adverse events occurred in our patients. All rituximab‐treated patients did not receive any premedication of steroids to avoid infusion reactions.

## Discussion

In the present study, three main findings were as follows. (1) In comparison with conventional therapy for CIDP, the better improvements and outcomes were quickly obtained by induction and maintenance treatment with combined low‐dose rituximab, thereby significantly reducing corticosteroids dosage and deterioration occurrence during the follow‐up. (2) The combination of low‐dose rituximab therapy presented the similarly favorable efficacy in both typical CIDP and CIDP variants. (3) Early initiating combined low‐dose rituximab treatment (<10 weeks) showed better improvements of CIDP in a short‐term than delayed initiation (≥10 weeks), while had no influence on the long‐term favorable prognosis of the regimen.

Recently, some researchers have found a persistent increasing memory B‐cell subset with lower inhibitory Fc gamma receptor IIB (FcγRIIB) expression in both typical CIDP and CIDP variants patients, which might fail to prevent memory B cells with self‐reactive receptors from entering the germinal center and becoming IgG‐positive plasma cells, thereby causing a chronic overactivity in the B‐cell mediated humoral immunity^31^, ^32^, ^33^. Given the dysregulation of B‐cell differentiation checkpoint might lower the activation threshold of memory B cells, thereby contributing to the pathogenesis of CIDP, a combined therapy of targeting effector memory B‐cell depletion should be helpful for better efficacy in CIDP.

Rituximab, initially approved for treatment in B‐cell lymphoma, is a human/murine chimeric monoclonal antibody targeting depletion of B lymphocytes by specific affinity to CD20, and gradually applied as an off‐label agent in many neuroimmune diseases treatment, due to covering immune‐dysfunctional memory B cells^15^, ^16^, ^17^, ^34^. The common high‐dose regimens of rituximab (375 mg/m^2^ or 1000 mg per infusion) were mainly derived from the experiences against high tumor burden of B‐cell lymphoma, which obviously seemed to be an overdosage for normal count but dysfunctional B cells in CIDP, usually exerting more medical expenses and serious adverse events with dose‐dependence^34^, ^35^, ^36^. However, there was still evidence in IgG4‐mediated MuSK MG relating the higher dosage with longer remission times^37^. Recently, low‐dose of 100 mg rituximab per infusion has been tried in some neuroimmune diseases, and the protocol consisted of infusion once a week for three cycles for induction, then followed by reinfusions at regular intervals for maintenance, which still showed sufficient depletion of B cells and favorable clinical outcomes with reduced side‐effect and cost^20^, ^21^, ^22^, ^23^, ^24^. Thus, in this study, we tried a simplified regimen of combined low‐dose rituximab in CIDP treatment.

During our follow‐up within 52 weeks, compared to control cohort with conventional therapy for CIDP, combined low‐dose rituximab cohort not only showed better comprehensive improvements and outcomes in a series of scales assessment (including I‐RODS, mRS, INCAT, ONLS, TSS, and COMPASS 31) at each visit, but also significantly reduced corticosteroids dosage and deterioration occurrence. Longitudinal analyses of intra‐ conventional therapy cohort indicated no differences from baseline at each visit. While in combined rituximab cohort, the proportions of favorable outcomes in clinical assessments for CIDP were quickly, continuously and significantly increased during the first 3 visits (within 28 weeks) after induction treatment with low‐dose rituximab, then maintained till the last visit (52 weeks) by a regular reinfusion at 28 weeks. Further analyses for subgroups revealed that, even with more severity at baseline, the typical CIDP and CIDP variants subgroup with combined rituximab treatment still presented better outcomes than subgroup with conventional therapy respectively, but had no differences in favorable efficacy between each other. Moreover, in comparison with delayed initiation (≥10 weeks) subgroup, early initiating combined rituximab regimen (<10 weeks) for CIDP presented better improvements at the first three visits within 28 weeks for a short‐term, while had no influence on the favorable long‐term prognoses of the regimen at the last visit of 52 weeks.

The following limitations should be considered in our study: (1) a retrospective analysis was designed without comparison with natural course of CIDP or other dosing regimens as control; (2) a small sample size with limited follow‐up time could not be completely avoid bias in selecting patients; (3) data were collected during routine clinical practice rather than a formal study setting, which meant quality and quantity varied among patients; (4) conventional treatments were performed irregularly in both control and combined rituximab cohort, which might cause undervaluation of the therapeutic effect in the control cohort. But had no influence on the evaluation of combined rituximab efficacy. Because of difficulties in randomized trials for rare neuroimmune diseases such as CIDP, real‐world data from clinical practice might contribute to important information on therapeutic profiles and protocols. Although limited data in our study, the result about efficacy and safety of combined low‐dose rituximab might be encouraging and presenting implications for CIDP treatment, and future prospective studies with a larger sample size are required.

Altogether, our simplified regimen of combined low‐dose rituximab has been demonstrated for the better efficacy and safety than conventional therapy in CIDP treatment.

## Author Contributions

Wei Zhang and Yingjun Zhao had full access to all the data in the study and takes full responsibility for the integrity of the data and the accuracy of the data analysis. Ying Du, Qi Yan, Chuan Li, and Wenping Zhu contributed equally to this study and are co‐first authors.

Study concept and design: Ying Du, Yingjun Zhao, and Wei Zhang. Acquisition, analysis, or interpretation of data: All authors. Drafting of the manuscript: Ying Du, Qi Yan, Yingjun Zhao, and Wei Zhang. Critical revision of the manuscript for important intellectual content: All authors. Statistical analysis: Ying Du, Chuan Li, and Wenping Zhu. Study supervision: Yingjun Zhao and Wei Zhang.

## Conflict of Interest

The authors declare that they have no conflict of interest in the submission of this manuscript, and the manuscript is approved by all the authors for publication.

## Supporting information


Figure S1.

Table S1.

Captions.

## Data Availability

The data that support the findings of this study are available from the corresponding author, upon reasonable request.
