# Supplementary material for: Efficacy and safety of combined low‐dose rituximab regimen for chronic inflammatory demyelinating polyradiculoneuropathy
Source: Ann Clin Transl Neurol. 2024 Dec 11;12(1):180–91. doi: 10.1002/acn3.52270 (PMC11752089; doi:10.1002/acn3.52270)
Supplement: Supplementary file 1 — Figure S1. [file ACN3-12-180-s003.pdf]

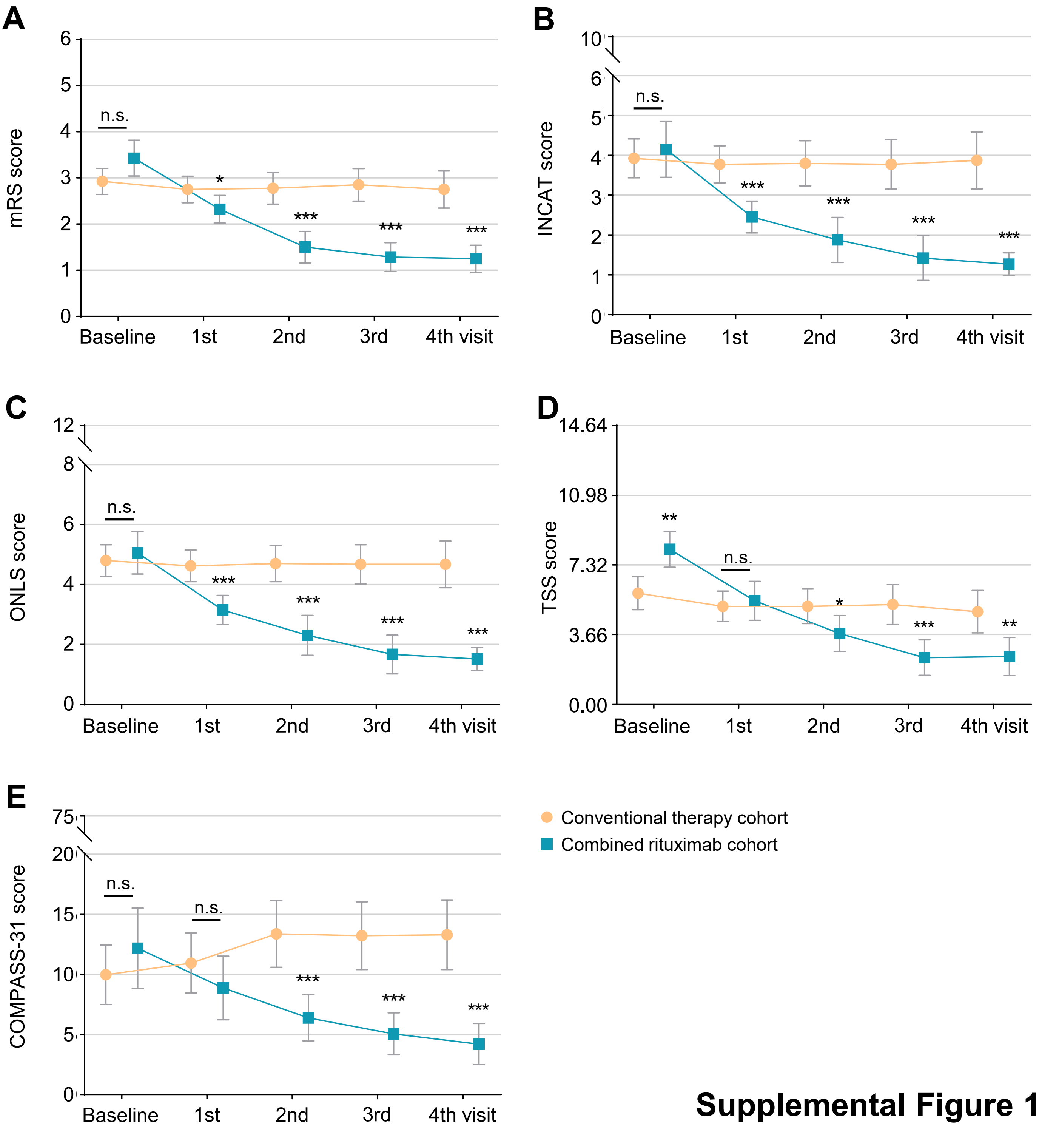

Supplemental Figure 1

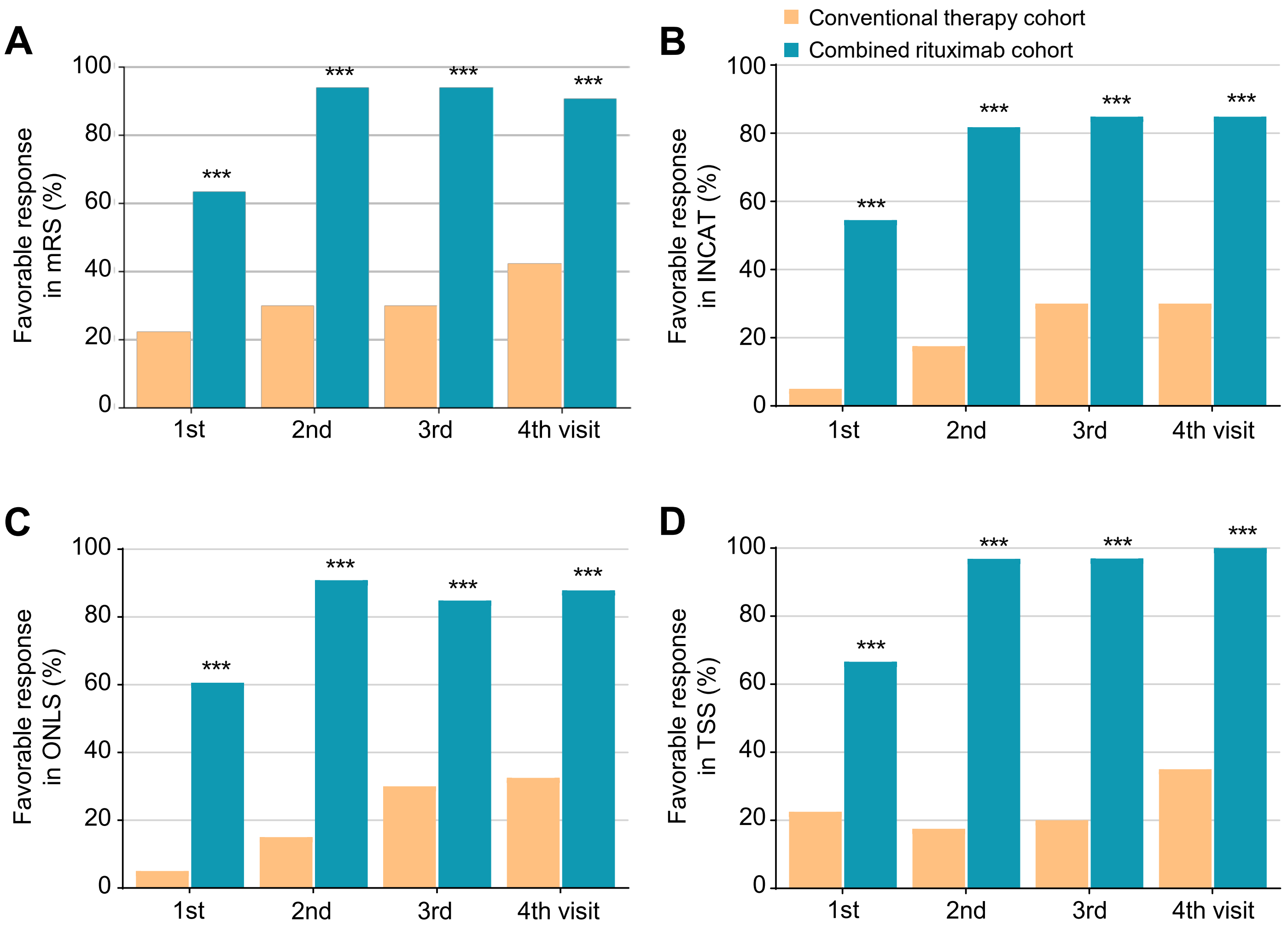

Supplemental Figure 2

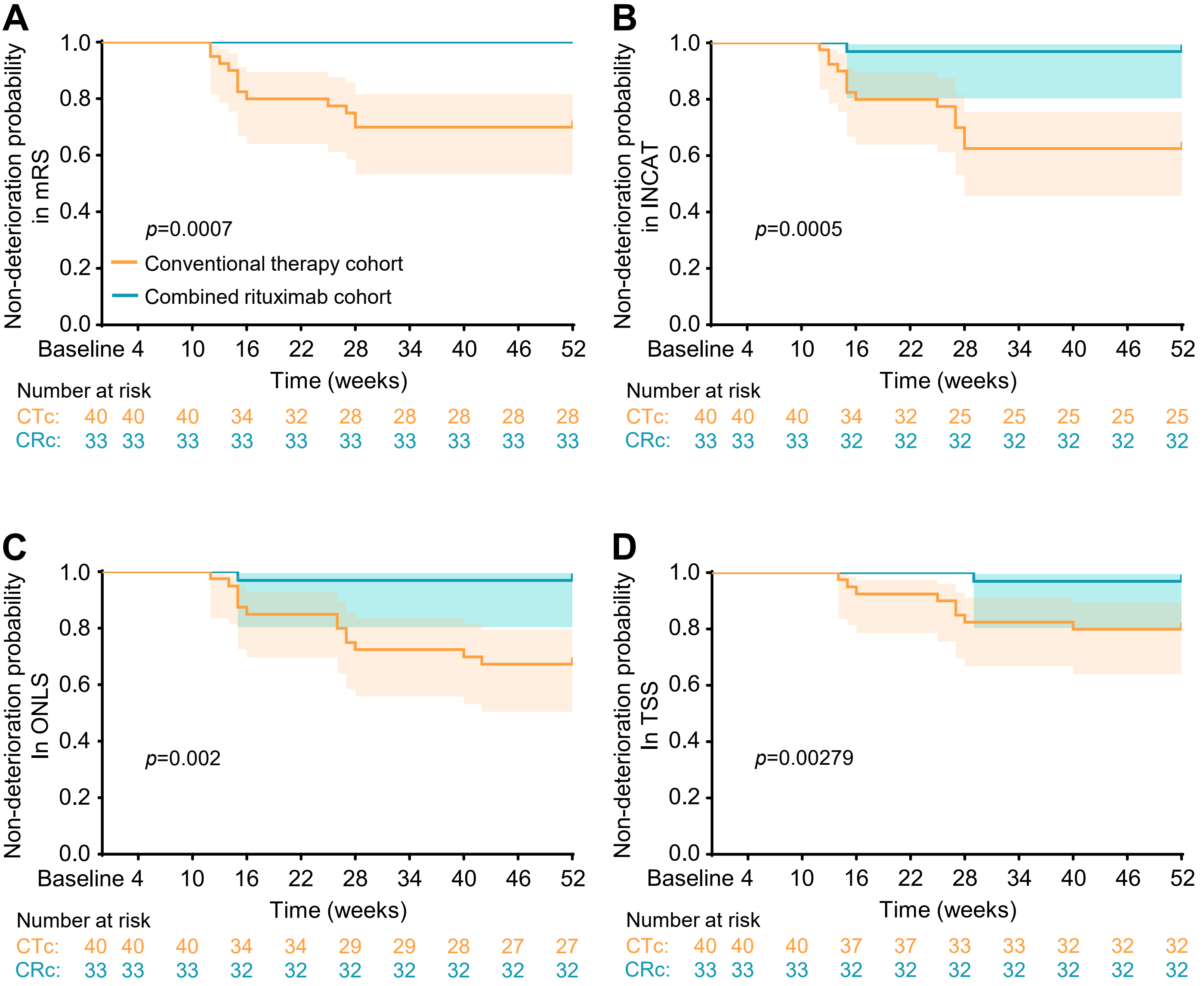

Supplemental Figure 3

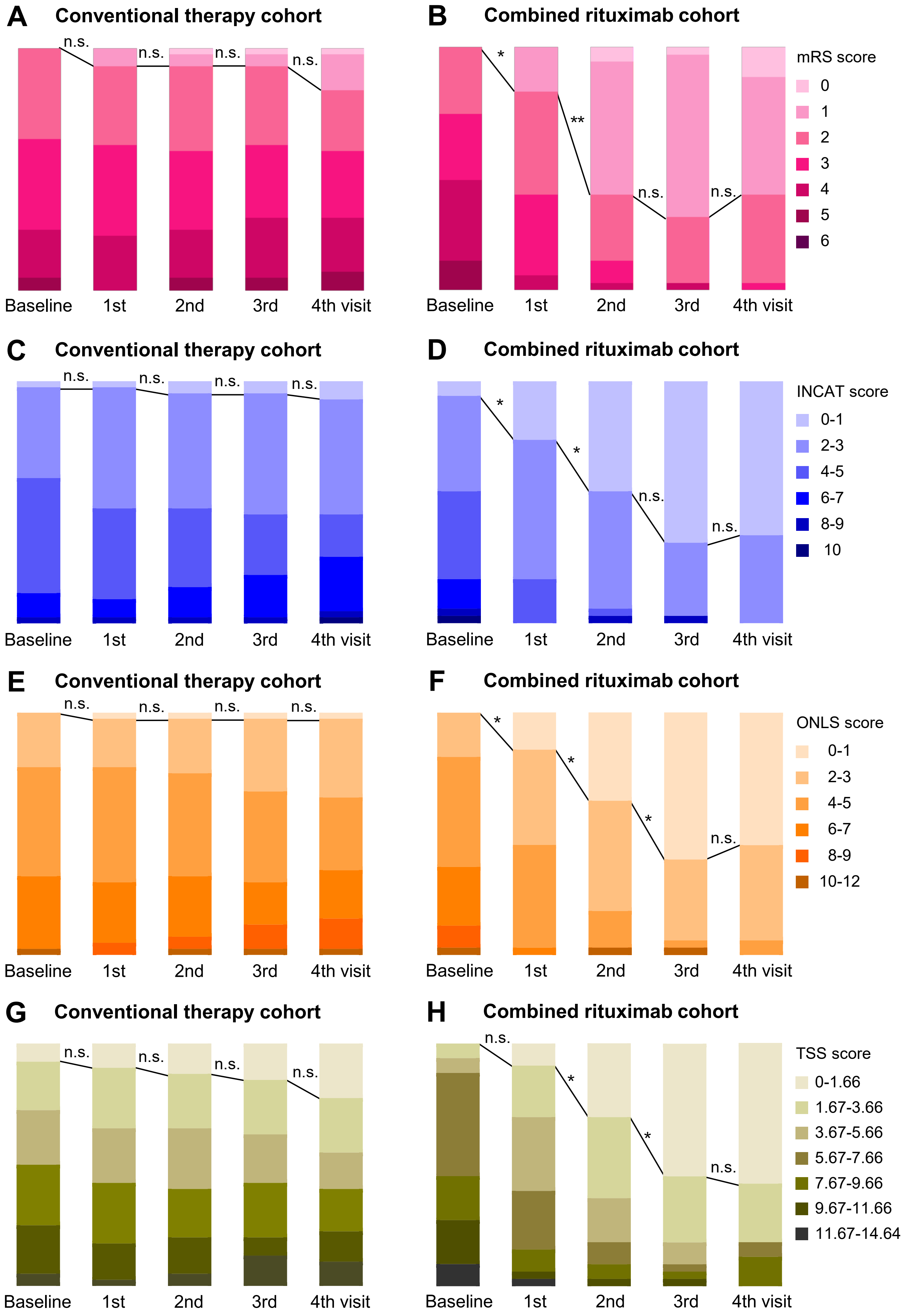

Supplemental Figure 4

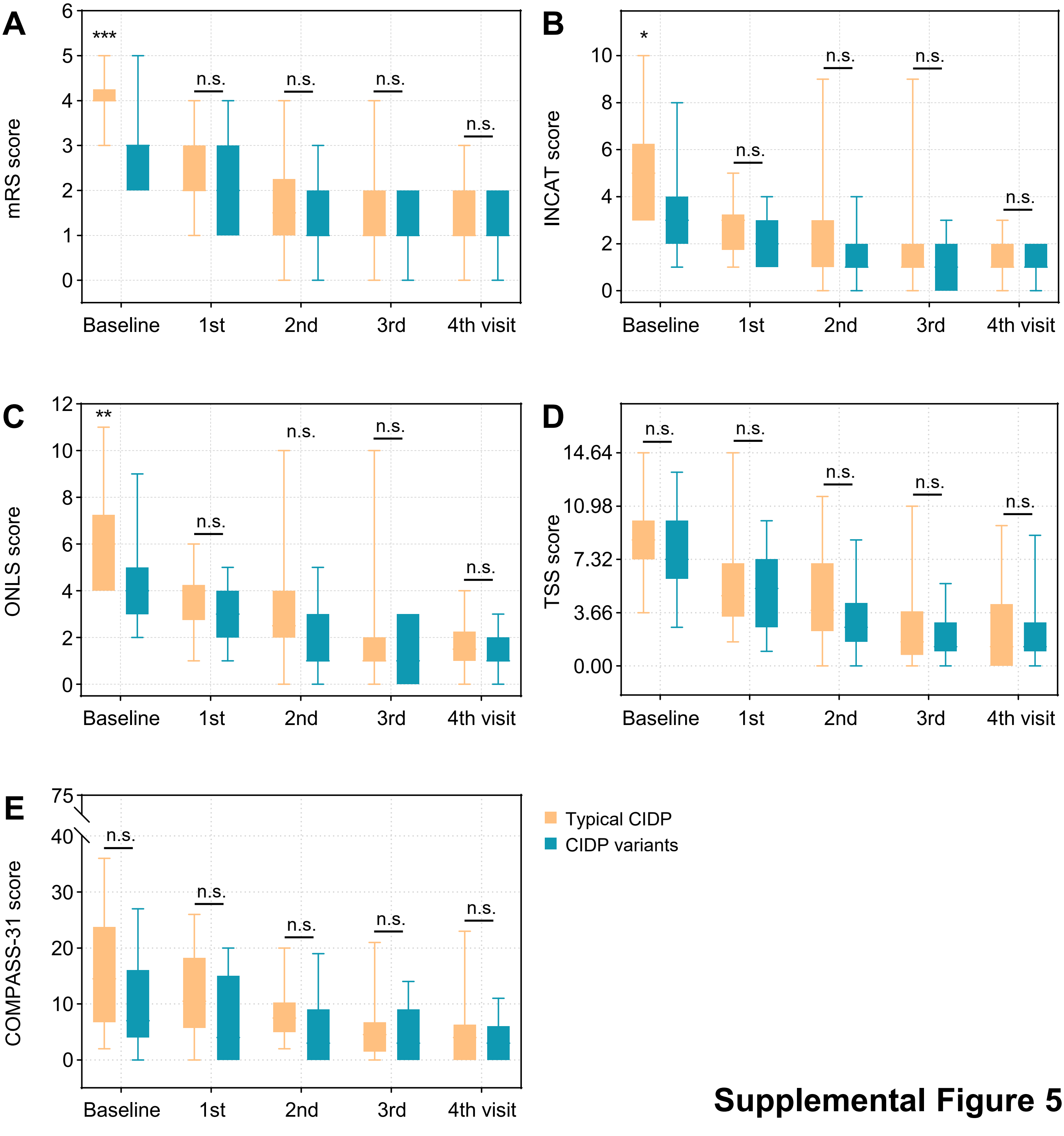

Supplemental Figure 5

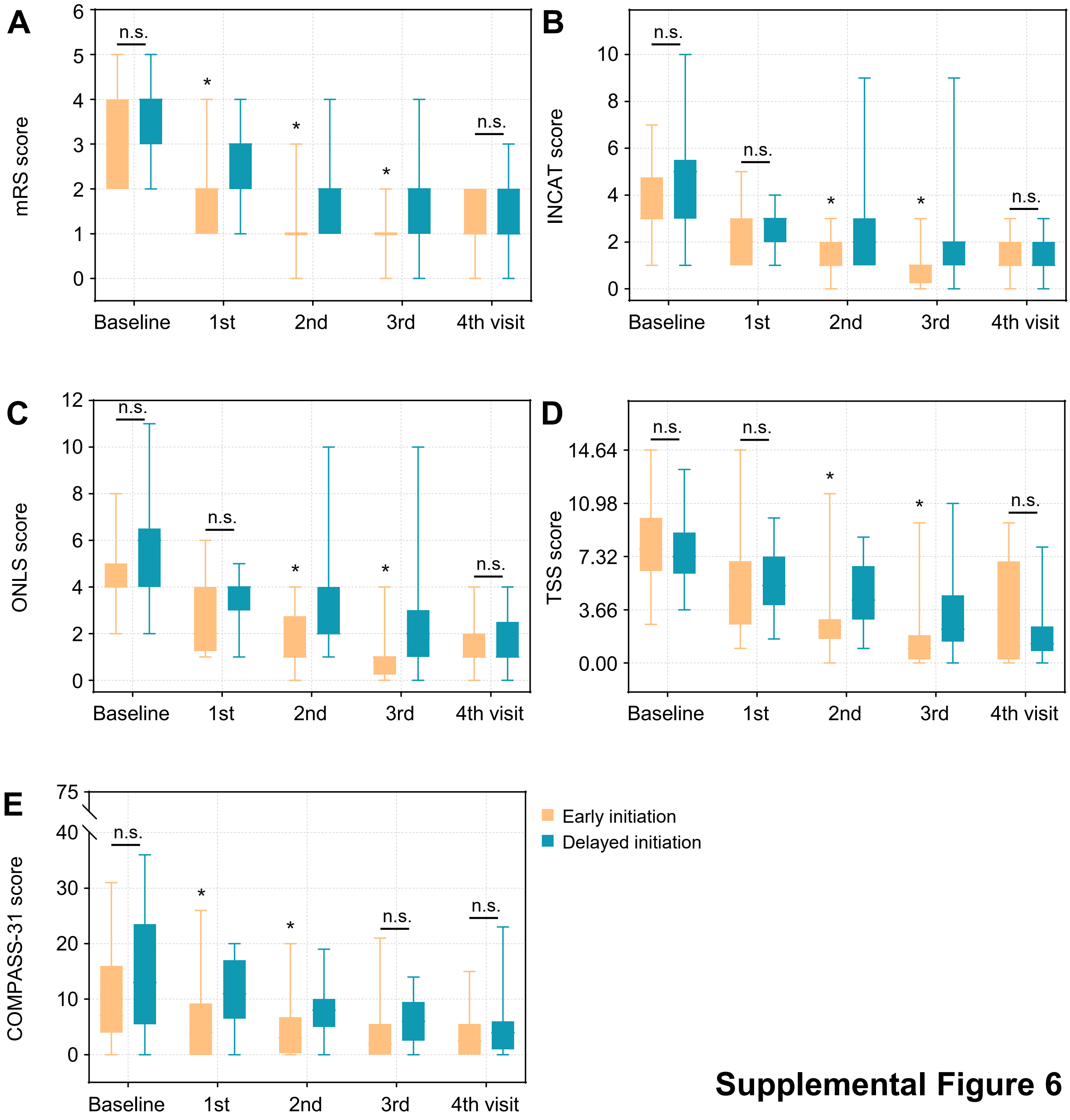

Supplemental Figure 6
