# Supplementary material for: Efficacy and safety of combined low‐dose rituximab regimen for chronic inflammatory demyelinating polyradiculoneuropathy
Source: Ann Clin Transl Neurol. 2024 Dec 11;12(1):180–91. doi: 10.1002/acn3.52270 (PMC11752089; doi:10.1002/acn3.52270)
Supplement: Supplementary file 2 — Table S1. [file ACN3-12-180-s001.pdf]

Supplemental Methods

Electrophysiologic studies

During the electrophysiologic studies, the room temperature should be maintained to ensure patients' limb skin temperature at least 33 °C on palm and 30 °C on external malleolus. Bilateral nerves were studied in all cases.

Motor NCSs were performed on the median, ulnar, radial, fibular, and tibial nerves with percutaneous supramaximal nerve stimulation. The median nerve was stimulated at the wrist, elbow, and axilla with recording from the abductor pollicis brevis; the ulnar nerve was stimulated at the wrist, below the elbow, above the elbow, and at the axilla with recording from the abductor digiti minimi.

Sensory NCSs were performed antidromically on the median, ulnar, superficial radial and sural nerves. The median and ulnar nerves were stimulated 3-cm proximal to the wrist crease with recording from digits II and V respectively; the radial and sural nerve were recorded over the anatomical snuff box and behind the lateral malleolus after stimulating 12- and 14-cm proximal to the active recording electrode respectively.

Cerebrospinal fluid analysis

According to the EFNS/PNS supportive criteria, the values of CSF protein >0.6 g/L above age 50 and ≥0.5 g/L under age 50 were set as cut-offs for elevation respectively, while the cytologic count >10/μL in CSF was defined as pleocytosis.

Statistical analysis

Missing values

In this study, we encountered missing values for certain variables in the baseline dataset: serum calcium (4 cases in the experimental group, 2 cases in the control group), cerebrospinal fluid biochemical and routine indicators (2 cases in the experimental group, 3 cases in the control group). No imputation was performed for these missing data. Notably, all other variables in our dataset were complete, with no missing values.

Sample Size Calculation

This trial used a differential trial design. The primary outcome was the treatment response rate. Assuming a response rate of 85% in the combined rituximab treatment group (experimental group) and 50% in the conventional first-line immunotherapy group (control

group), two-sided test, α=0.05, β=0.1 (i.e. 90% power), the trial group and the control group were assigned in a 1:1 ratio. The required sample size for each group was 33 cases, calculated by Power Analysis and Sample Size Software (PASS) Version 15.0 (NCSS, LLC, Kaysville, UT, USA). It is worth emphasizing that in accordance with the inclusion and exclusion criteria, the experimental group enrolled 33 patients, while the control group enrolled 40 patients during the same period, totaling 73 subjects.

Supplemental Table 1. Scales of CIDP for clinical outcome measures.

Outcome	Measures	Evaluation items	Range
**Primary outcome**			
**Disability**	Inflammatory Rasch-Overall Disability Scale (I-RODS) [1,2]	Washing your upper body; dressing your upper body; brush your teeth; move a chair; go to the toilet; make a sandwich; washing your lower body; eating; catch an object; turn a key in a lock; go to the general practitioner; read a newspaper or book; take a shower; do the washing up; walking while avoiding obstacles; walk one flight of stairs; bend and pick up an object; do the shopping; travel by public transportation; walk outdoors, up to max 0.6 mile; carry and put down a heavy object; remain standing for a long period of time; dance; run.	(0-48 points or 0-100 centile points) Higher scores represent lower levels of disability.
**Secondary outcomes**			
**Functional recovery**	The Modified Rankin Scale (mRS) [3]	The mRS was first introduced in 1957 for measuring outcomes in acute stroke[4] and was modified by Charles Warlow and others as part of the UK-TIA trial in the 1980s[5] and its reproducibility was first examined by van Swieten, et al., in 1988[3]. The current version as follows: 0, no symptoms; 1, no significant disability; 2, slight disability; 3, moderate disability; 4, moderately severe disability; 5, severe disability; and 6, dead.	(0-6 points) From perfect health without symptoms to death.
**Both upper and lower limbs disability**	Inflammatory Neuropathy Cause and Treatment disability scale (INCAT) [6]	The upper limb score is assigned as 0–5 based on whether the patient rates the following 4 tasks as “not affected,” “affected but not prevented,” or “prevented”: doing all buttons and zippers, washing or brushing hair, turning a key in a lock, and handling small coins. The lower limb score is assigned as 0–5 based on whether mobility is rated as: “walks without difficulty,” “walks independently, with difficulty,” “requires 1 cane,” “requires 2 canes / a walker,” “requires a wheelchair but can walk a few steps,” or “confined to wheelchair.”	(0-10 points) Higher scores represent lower levels of limb dysfunction.
**Activities of daily living**	Overall Neuropathy Limitation Scale (ONLS) [7]	Arm Grade: Wash and brush their hair; Turn a key in a lock; Use a knife and fork together; Do or undo buttons or zips; Dress the upper part of their body excluding buttons or zips. Leg grade: Does the patient have difficulty running or climbing stairs? Does the patient have difficulty with walking? Does their gait look abnormal? How do they mobilize for about 10 metres (ie 33 feet)? Without aid,	(0-12 points) Higher scores represent lower levels of activities measure.

		With one stick or crutch or holding to someone's arm, With two sticks or crutches or one stick or, crutch holding onto, someone's arm or frame, With a wheelchair; If they use a wheelchair, can they stand and walk 1 metre with the help of one person? If they cannot walk as above, are they able to make some purposeful movements of their legs, eg reposition legs in bed? Does the patient use ankle foot orthoses/braces?	
**Global sensory symptoms**	Total Symptom Score (TSS) [8]	It is a questionnaire in which the patient is asked to assess the intensity (absent, mild, moderate, severe). Moreover, frequency (now and then, often, continuous) of four symptoms (pain, burning, paresthesia, numbness) is also assessed resulting in a scaled score in which 0 means no symptoms and 14.64 means that all four symptoms are severe and continuously happened.	(0-14.64 points) Ranged from no to maximum symptoms.
**Autonomic symptoms**	Composite Autonomic Symptom Score-31 (COMPASS-31) [9]	The questions comprising this instrument address 11 autonomic domains with 72 questions (orthostatic intolerance, 9 items; secretomotor, 8 items; male sexual dysfunction, 8 items; urinary, 3 items; gastroparesis, 5 items; constipation, 4 items; diarrhea, 4 items; pupillomotor, 7 items; vasomotor, 11 items; reflex syncope, 5 items; and sleep, 8 items) and an additional 12 items to generate 2 validity scores (an understatement index comprising 6 questions and a psychosomatic index comprising 6 questions).	(0-75 points or 0-100 centile points) higher scores indicating a more severe level of autonomic symptoms dysfunction.

References

1. van Nes SI, Vanhoutte EK, van Doorn PA, et al. Rasch-built Overall Disability Scale (R-ODS) for immune-mediated peripheral neuropathies. *Neurology.* 2011;76(4):337-345.
2. Peric S, Bozovic I, Pruppers MHJ, et al. Validation of the Serbian version of inflammatory Rasch-built overall disability scale in patients with chronic inflammatory demyelinating polyradiculoneuropathy. *J Peripher Nerv Syst.* 2019;24(3):260-267.
3. van Swieten JC, Koudstaal PJ, Visser MC, Schouten HJ, van Gijn J. Interobserver agreement for the assessment of handicap in stroke patients. *Stroke.* 1988;19(5):604-607.
4. Quinn TJ, Dawson J, Walters M. Dr John Rankin; his life, legacy and the 50th anniversary of the Rankin Stroke Scale. *Scott Med J.* 2008;53(1):44-47.
5. Farrell B, Godwin J, Richards S, Warlow C. The United Kingdom transient ischaemic attack (UK-TIA) aspirin trial: final results. *J Neurol Neurosurg Psychiatry.* 1991;54(12):1044-1054.
6. Breiner A, Barnett C, Bril V. INCAT disability score: a critical analysis of its measurement properties. *Muscle Nerve.* 2014;50(2):164-169.
7. Graham RC, Hughes RA. A modified peripheral neuropathy scale: the Overall Neuropathy Limitations

Scale. *J Neurol Neurosurg Psychiatry.* 2006;77(8):973-976.

8. Ziegler D, Hanefeld M, Ruhnau KJ, et al. Treatment of symptomatic diabetic peripheral neuropathy with the anti-oxidant alpha-lipoic acid. A 3-week multicentre randomized controlled trial (ALADIN Study). *Diabetologia.* 1995;38(12):1425-1433.
9. Suarez GA, Opfer-Gehrking TL, Offord KP, Atkinson EJ, O'Brien PC, Low PA. The Autonomic Symptom Profile: a new instrument to assess autonomic symptoms. *Neurology.* 1999;52(3):523-528.

Supplemental Table 2. Comparison of electrophysiologic studies between conventional therapy and combined rituximab cohort at baseline.

	CT Cohort (n=40) Mean, Median (min - max)	CR Cohort (n=33) Mean, Median (min - max)	*P* Value
**MCV Number**	n=91	n=80	
**No. of nerve injuries**	2.275, 0 (0 - 10)	2.424, 0 (0 - 10)	.835
**Peroneal. R**	13, 0 (0 - 37)	5.62, 0 (0 - 37)	.412
**Peroneal. L**	9.61, 0 (0 - 37)	5.59, 0 (0 - 37)	.424
**Tibial. R**	19.7, 24.3 (0 - 39)	12, 0 (0 – 36.47)	.332
**Tibial. L**	18.9, 23 (0 - 41)	10.57, 0 (0 - 36)	.256
**Median. R**	28.25, 32.3(0 - 47)	18.42, 21 (0 - 49)	.195
**Median. L**	27.5, 33.3(0 - 45)	21.23, 22.45 (0 - 48)	.529
**Ulnar. R**	27, 29 (0 – 54.9)	21.55, 21 (0 - 45)	.469
**Ulnar. L**	21.5, 20 (0 – 54.8)	20.36, 19.5 (0 - 48)	.805
**Radial. R**	28.32, 24.45 (20 - 44.4)	9.25, 0 (0 - 37)	.139
**Radial. L**	26.85, 26 (13.6 - 41.8)	7.5, 0 (0 - 30)	.139
**CMAP Number**	n=154	n=130	
**No. of nerve injuries**	3.85, 4 (0 - 12)	4.06, 3.5 (0 - 12)	.686
**Peroneal. R**	0.8, 0.57 (0 - 2.7)	0.47, 0.3 (0 - 1.8)	.248
**Peroneal. L**	0.9, 0.8 (0 - 3)	0.51, 0.36 (0 - 1.7)	.282
**Tibial. R**	0.78, 0.5 (0 - 3.3)	1.08, 0.45 (0 - 8.3)	.919
**Tibial. L**	1.69, 1.4 (0 - 8.8)	0.72, 0.5 (0 - 3.1)	.109
**Femoral. R**	1.53, 1.05 (0 - 3.8)	1.53, 1.1 (0 - 4.7)	.697
**Femoral. L**	1.4, 0.64 (0 - 3.8)	1.27, 4 (0 - 3.7)	.631
**Median. R**	1.85, 1.9 (0 - 3.5)	1, 0.4 (0 - 3.1)	.062
**Median. L**	2.07, 2.1 (0 - 4.2)	0.72, 0.4 (0 - 2.7)	.036
**Ulnar. R**	3.6, 3.05 (0 - 10.3)	1.32, 0.4 (0 - 4.4)	.056
**Ulnar. L**	3.66, 2.7 (0 - 11.3)	0.87, 0.6 (0 - 2.4)	.005
**Radial. R**	1.97, 1.75 (1 - 3.4)	0.84, 0.4 (0 - 2.6)	.127
**Radial. L**	2.31, 3 (0.73 - 3)	0.63, 0.1 (0 - 2.1)	.066
**SCV Number**	n=184	n=158	
**No. of nerve injuries**	4.6, 4 (0 - 10)	4.78, 4 (0 - 10)	.845
**Superficial peroneal. R**	0	0	NA
**Superficial peroneal. L**	0	0	NA
**Sural. R**	0	0	NA
**Sural. L**	0	0	NA
**Median. R**	11.72, 0 (0 - 49.8)	7.55, 0 (0 - 42.1)	.559
**Median. L**	6.53, 0 (0 - 49)	7.68, 0 (0 - 43.5)	.858
**Ulnar. R**	2.78, 0 (0 - 36.2)	7.86, 0 (0 - 45)	.392

**Ulnar. L**	2.96, 0 (0 - 35.6)	4.55, 0 (0 - 45.5)	.843
**Radial. R**	4.82, 0 (0 - 33.8)	0	.398
**Radial. L**	4.95, 0 (0 - 34.7)	0	.398
**SNAP Number**	n=241	n=178	
**No. of nerve injuries**	6.02, 6.5 (0 - 10)	5.39, 4 (0 - 10)	.425
**Superficial peroneal. R**	0.21, 0 (0 - 6.9)	0.4, 0 (0 - 10.9)	.884
**Superficial peroneal. L**	0.19, 0 (0 - 6.3)	0	.397
**Sural. R**	0.12, 0 (0 - 3.6)	0	.361
**Sural. L**	0.21, 0 (0 - 6.5)	0	.360
**Median. R**	7.66, 5.15 (0 - 31.8)	2.59, 0 (0 - 12.2)	.126
**Median. L**	8.89, 4.6 (0 - 37.4)	3.09, 0 (0 - 14.8)	.118
**Ulnar. R**	4.24, 0 (0 - 16.7)	2.15, 0 (0 - 9.1)	.385
**Ulnar. L**	4.12, 2.45 (0 - 17.1)	3.58, 0 (0 - 36.5)	.264
**Radial. R**	3.88, 0 (0 - 22.5)	0	.174
**Radial. L**	1.96, 0 (0 - 12.9)	1, 0 (0 - 7)	.769

Abbreviations: CT Cohort: Conventional Therapy Cohort; CR Cohort: Combined Rituximab Cohort; L: left; R: right; MCV: motor conduction velocity; CMAP: compound muscle action potential; SCV: sensory conduction velocity; SNAP: sensory nerve action potential.

Supplemental Table 3. Comparison of laboratory data in blood and CSF between conventional therapy and combined rituximab cohort at baseline.

Parameter	CT Cohort median (IQ range)	n	CR Cohort median (IQ range)	n	*P* Value	Normal range (units)
**Blood**						
**ALA**	23.5(15.5)	40	21(20.5)	33	0.415	(Female: 11-66; Male: 9-50) U/L
**BIL**	13.6(7.75)	40	15.37(6.05)	33	0.510	(Female: 3-22; Male: 0-26) μmol/L
**BUN**	4.9(2.38)	40	5.2(2.45)	33	0.272	(2.9-8.2) mmol/L
**SCR**	56.5(16.75)	40	50(12.5)	33	0.292	(Female: 28-110; Male: 51-111) μmol/L
**BG**	5.15(0.81)	40	5.15(1.19)	33	0.295	(3.9-6.1) mmol/L
**Potassium**	3.9(0.5)	40	3.83(0.5)	33	0.428	(3.5-5.5) mmol/L
**Sodium**	140.75(2.7)	40	140.8(2.45)	33	0.575	(135-145) mmol/L
**Calcium**	2.3(0.15)	36	2.28(0.12)	31	0.345	(2.25-2.75) mmol/L
**WBC**	5.34(2.37)	40	5.14(2.21)	33	0.973	(3.5-9.5) $\times10^{9}$
**PLT**	192.5(69.5)	40	195(65.5)	33	0.576	(100-300) $\times10^{9}$
**RBC**	4.27(0.8)	40	4.26(0.55)	33	0.769	(Female: 3.5-5; Male: 4-5.5) $\times10^{12}$
**HCT**	41.4(8.52)	40	39.9(5.65)	33	0.265	(Female: 38-50.8; Male: 33.5-45) %
**HGB**	137(28.75)	40	130(19.5)	33	0.265	(Female: 113-151; Male: 131-172) g/L
**CSF**						
**Pressure**	140(45)	37	130(42.5)	30	0.681	(100-180) mmH_2O
**Leukocytes**	0(4)	37	0(2.5)	30	0.453	(0-8) X 10^6/L
**Chloride**	126.25(3.92)	38	126.35(3.17)	30	0.521	(120-130) mmol/L
**Glucose**	3.81(0.92)	38	3.88(0.97)	30	0.739	(2.5-4.5) mmol/L
**TP**	386.28(299.58)	38	464.95(435.85)	30	0.142	(Age ≥50 y: 150-600; <50 y: 150-500) mg/L

Abbreviations: CT Cohort: Conventional Therapy Cohort; CR Cohort: Combined Rituximab Cohort; ALA: alanine; BIL: bilirubin; BUN: blood urea nitrogen; SCR: serum creatinine; BG: blood glucose; WBC: white blood cells; PLT: platelets; RBC: red blood cells; HCT: hematocrit; HGB: hemoglobin; CSF: cerebrospinal fluid; TP: total protein.

Supplemental Table 4. Longitudinal intra-cohort analyses for improvements in conventional therapy and combined rituximab cohort.

	Evaluation scale	Baseline Median (IQR)	1st visit Median (IQR)	*P* Value[a]	2nd visit Median (IQR)	*P* Value[a]	3rd visit Median (IQR)	*P* Value[a]	4th visit Median (IQR)	*P* Value[a]
CT Cohort (n=40)	**I-RODS**	55(20)	60(19)	.90	56(21.5)	.99	55(24.5)	.99	56(35.5)	.89
	**mRS**	3(1.75)	3(1)	.37	3(1.75)	.42	0(2)	.69	0(1.75)	.46
	**INCAT**	4(2)	3(2)	.59	3(2)	.63	3(3)	.47	3(4)	.56
	**ONLS**	5(2)	4(2)	.57	4(2.75)	.69	4(3)	.54	4(4)	.54
	**TSS**	5.6(4.24)	5.32(3)	.17	5.32(4.24)	.19	5.3(4.3)	.26	4.82(5.32)	.10
	**COMPASS-31**	9.5(10.5)	10(10)	.47	13.5(13.75)	.07	11.5(16)	.08	12(14.25)	.07
CR Cohort (n=33)	**I-RODS**	52(19)	65(15.5)	***<.001***	76(20)	***<.001***	88(15)	***<.001***	88(16)	***<.001***
	**mRS**	3(2)	2(1)	***<.001***	1(1)	***<.001***	1(1)	***<.001***	1(1)	***<.001***
	**INCAT**	4(2)	2(1.5)	***<.001***	2(1)	***<.001***	1(1)	***<.001***	1(1)	***<.001***
	**ONLS**	5(2)	3(2)	***<.001***	2(2)	***<.001***	1(1)	***<.001***	1(1)	***<.001***
	**TSS**	7.32(3.6)	5.32(4.1)	***<.001***	2.99(3.1)	***<.001***	1.66(1.8)	***<.001***	1.33(2.1)	***<.001***
	**COMPASS-31**	9(13.5)	7(13.5)	.18	6(7.5)	***.01***	4(7.5)	***.001***	3(6)	***<.001***

Abbreviations: CT Cohort: Conventional Therapy Cohort; CR Cohort: Combined Rituximab Cohort; I-RODS, Inflammatory Rasch-Overall Disability Scale; mRS, modified Rankin Scale; INCAT, Inflammatory Neuropathy Cause and Treatment disability scale; ONLS, Overall Neuropathy Limitation Scale; TSS, Total Symptom Score; COMPASS-31, Composite Autonomic Symptom Score-31.

[a]Comparison between baseline and each visit.

Supplemental Table 5. Longitudinal intra-subgroup analyses of different treatment in conventional therapy cohort.

Treatment	Evaluation scale	Baseline Median (IQR)	1st visit Median (IQR)	*P* Value[a]	2nd visit Median (IQR)	*P* Value[a]	3rd visit Median (IQR)	*P* Value[a]	4th visit Median (IQR)	*P* Value[a]
**IVIG (n=9)**	I-RODS	60(27)	55(29	.95	47(36)	.10	43(49)	.99	42(51)	.10
	mRS	2(2)	3(2)	.75	3(2.5)	.82	3(2.5)	.75	3(2.5)	.79
	INCAT	4(2)	4(2)	.75	4(2.5)	.83	5(4.5)	.86	5(8)	.50
	ONLS	5(2.5)	5(2.5)	.82	5(3)	.79	6(5)	.89	6(5)	.62
	TSS	5.32(2.83	6.32(3)	.89	5.66(3.2)	.75	5.66(3.6)	.66	3.32(4.1)	.53
	COMPASS-31	8(8.5)	8(10.5)	.86	6(17)	.83	4(17.5)	.86	5(20.5)	.97
**CSs (n=27)**	I-RODS	55(17)	60(13	.99	57(17)	.99	55(17)	.99	61(25)	.89
	mRS	3(1)	3(1)	.78	3(5)	.82	3(2)	.93	3(1)	.27
	INCAT	4(2)	3(2)	.82	3(3)	.97	3(2)	.40	3(3)	.29
	ONLS	5(2)	4(2)	.83	4(3)	.97	4(2)	.45	4(3)	.32
	TSS	5.32(4.99)	5.32(3)	.26	4.9(4.3)	.29	4.99(4.9)	.33	4.32(6.6)	.10
	COMPASS-31	10(10)	10(11)	.75	13(12)	.08	10(11)	.16	12(10)	.112
**Double treatments (n=4)**	I-RODS	41(30.25)	58.5(22.5)	.87	58(29.3)	.95	54.5(35.5)	.96	48.5(43.25)	.99
	mRS	3.5(1.75)	2.5(1.7)	.45	2(3)	.37	2.5(2.5)	.37	3.5(2.5)	.77
	INCAT	4.5(2.5)	3.5(1.8)	.37	3(3)	.37	3.5(4)	.31	4(5)	.77
	ONLS	5.5(3.25)	4.5(2.5)	.46	4(3)	.5	4.5(4)	.47	5(5.7)	.89
	TSS	7.32(4.99)	6.32(5.4)	.77	7.32(4.9)	.278	7.32(7.4)	.22	6.65(8.5)	.77
	COMPASS-31	12(12.75)	15.5(4)	.38	20(12)	.56	22.5(12.5)	.47	22.5(20.5)	.15

Abbreviations: IVIG: Intravenous immunoglobulin; CSs: Corticosteroids; I-RODS: Inflammatory Rasch-Overall Disability Scale; mRS: modified Rankin Scale; INCAT, Inflammatory Neuropathy Cause and Treatment disability scale; ONLS, Overall Neuropathy Limitation Scale; TSS, Total Symptom Score; COMPASS-31, Composite Autonomic Symptom Score-31.

[a] Comparison between baseline and each visit.

Supplemental Table 6. Longitudinal intra-subgroup analyses of different CIDP subtype in conventional therapy cohort.

Subtype	Evaluation scale	Baseline Median (IQR)	1st visit Median (IQR)	*P* Value[a]	2nd visit Median (IQR)	*P* Value[a]	3rd visit Median (IQR)	*P* Value[a]	4th visit Median (IQR)	*P* Value[a]
**Typical CIDP (n=23)**	I-RODS	57(18)	60(18)	.98	58(21)	.97	55(23)	.98	58(37)	.99
	mRS	3(2)	3(1)	.98	3(1)	.82	3(2)	.89	3(2)	.89
	INCAT	4(2)	4(2)	.99	3(1)	.98	4(2)	>.99	3(4)	.99
	ONLS	5(2)	5(2)	.99	4(2)	.98	5(2)	>.99	4(4)	>.99
	TSS	6.32(4.7)	6.32(4.9)	.91	5.32(4.9)	.89	5.66(5.7)	.96	5.65(6.7)	.86
	COMPASS-31	8(12)	10(10)	.88	15(10)	.28	13(11)	.27	13(11)	.19
**CIDP Variants (n=17)**	I-RODS	52(22)	57(23)	.89	55(25)	.99	55(31.5)	>.99	55(36)	.81
	mRS	3(1.5)	3(1.5)	.91	3(2)	>.99	3(2)	.99	3(1.5)	.99
	INCAT	4(2)	3(2)	.97	4(2.5)	>.99	3(4)	.91	3(4)	.97
	ONLS	5(2.5)	4(2.5)	.98	5(3)	>.99	4(4)	.96	4(4.5)	.96
	TSS	5.32(3.7)	5.32(3)	.81	4.66(3.2)	.84	4(3.3)	.84	3.32(4.5)	.40
	COMPASS-31	10(8.5)	10(10.5)	>.99	10(16)	.83	9(16)	.91	10(13)	.96

Abbreviations: CIDP: chronic inflammatory demyelinating polyradiculoneuropathy; I-RODS, Inflammatory Rasch-Overall Disability Scale; mRS, modified Rankin Scale; INCAT, Inflammatory Neuropathy Cause and Treatment disability scale; ONLS, Overall Neuropathy Limitation Scale; TSS, Total Symptom Score; COMPASS-31, Composite Autonomic Symptom Score-31.

[a] Comparison between baseline and each visit.

Supplemental Table 7. Transversal inter-subgroup analyses among different treatments in conventional therapy cohort.

Evaluation scale, median (IQR)	Baseline				1st visit				2nd visit				3rd visit				4th visit			
	**IVIG (n=9)**	**CSs (n=27)**	**Double (n=4)**	***P* Value**	**IVIG (n=9)**	**CSs (n=27)**	**Double (n=4)**	***P* Value**	**IVIG (n=9)**	**CSs (n=27))**	**Double (n=4)**	***P* Value**	**IVIG (n=9)**	**CSs (n=27)**	**Double (n=4)**	***P* Value**	**IVIG (n=9)**	**CSs (n=27)**	**Double (n=4)**	***P* Value**
**I-RODS**	60(27)	55(17)	41(30.3)	.29	55(29)	60(13)	58.5(22.5)	.86	47(36)	57(17)	58(29.3)	.61	43(49)	55(17)	54.5(35.5)	.63	42(51)	61(25)	48.5(43.25)	.35
**mRS**	2(2)	3(1)	3.5(1.7)	.75	3(2)	3(1)	2.5(1.7)	.75	3(2.5)	3(5)	2(3)	.94	3(2.5)	3(2)	2.5(2.5)	.95	3(2.5)	3(1)	3.5(2.5)	.30
**INCAT**	4(2)	4(2)	4.5(2.5)	.42	4(2)	3(2)	3.5(1.8)	.74	4(2.5)	3(3)	3(3)	.55	5(4.5)	3(2)	3.5(4)	.18	5(8)	3(3)	4(5)	.11
**ONLS**	5(2.5)	5(2)	5.5(3.3)	.65	5(2.5)	4(2)	4.5(2.5)	.87	5(3)	4(3)	4(3)	.69	6(5)	4(2)	4.5(4)	.29	6(5)	4(3)	5(5.7)	.23
**TSS**	5.32(2.8)	5.32(4.9)	7.32(4.9)	.54	6.32(3)	5.32(3)	6.32(5.4)	.74	5.66(3.2)	4.9(4.3)	7.32(4.9)	.58	5.66(3.6)	4.99(4.9)	7.32(7.4)	.78	3.32(4.1)	4.32(6.6)	6.65(8.5)	.74
**COMPASS-31**	8(8.5)	10(10)	12(12.7)	.67	8(10.5)	10(11)	15.5(4)	.33	6(17)	13(12)	20(12)	.36	4(17.5)	10(11)	22.5(12.5)	.07	5(20.5)	12(10)	22.5(20.5)	.07

Abbreviations: IVIG: Intravenous immunoglobulin; CSs: Corticosteroids; I-RODS, Inflammatory Rasch-Overall Disability Scale; mRS, modified Rankin Scale; INCAT, Inflammatory Neuropathy Cause and Treatment disability scale; ONLS, Overall Neuropathy Limitation Scale; TSS, Total Symptom Score; COMPASS-31, Composite Autonomic Symptom Score-31.

Supplemental Table 8. Transversal inter-subgroup analyses between CIDP subtypes in conventional therapy cohort.

Evaluation scale, median (IQR)	Baseline			1st visit			2nd visit			3rd visit			4rd visit		
	Typical CIDP (n=23)	CIDP Variants (n=17)	*P* Value	Typical CIDP (n=23)	CIDP Variants (n=17)	*P* Value	Typical CIDP (n=23)	CIDP Variants (n=17)	*P* Value	Typical CIDP (n=23)	CIDP Variants (n=17)	*P* Value	Typical CIDP (n=23)	CIDP Variants (n=17)	*P* Value
**I-RODS**	57(18)	52(22)	.27	60(18)	57(23)	.68	58(21)	55(25)	.26	55(23)	55(31.5)	.38	58(37)	55(36)	.63
**mRS**	3(2)	3(1.5)	.59	3(1)	3(1.5)	.96	3(1)	3(2)	.27	3(2)	3(2)	.29	3(2)	3(1.5)	.66
**INCAT**	4(2)	4(2)	.68	4(2)	3(2)	.86	3(1)	4(2.5)	.30	4(2)	3(4)	.57	3(4)	3(4)	.96
**ONLS**	5(2)	5(2.5)	.75	5(2)	4(2.5)	.88	4(2)	5(3)	.37	5(2)	4(4)	.46	4(4)	4(4.5)	.86
**TSS**	6.32(4.7)	5.32(3.7)	.74	6.32(4.9)	5.32(3)	.44	5.32(4.9)	4.66(3.2)	.53	5.66(5.7)	4(3.3)	.47	5.65(6.7)	3.32(4.5)	.35
**COMPASS-31**	8(12)	10(8.5)	.81	10(10)	10(10.5)	.50	15(10)	10(16)	.57	13(11)	9(16)	.25	13(11)	10(13)	.18

Abbreviations: I-RODS: Inflammatory Rasch-Overall Disability Scale; mRS: modified Rankin Scale; INCAT: Inflammatory Neuropathy Cause and Treatment disability scale; ONLS: Overall Neuropathy Limitation Scale; TSS: Total Symptom Score; COMPASS-31, Composite Autonomic Symptom Score-31.

Supplemental Table 9. Comparison of clinical outcomes in CIDP subtype between conventional therapy cohort and combined rituximab cohort.

CIDP Subtype	Evaluation scale, median (IQR)	Baseline			1st visit			2nd visit			3rd visit			4th visit		
		CT Cohort	CR Cohort	*P* Value	CT Cohort	CR Cohort	*P* Value	CT Cohort	CR Cohort	*P* Value	CT Cohort	CR Cohort	*P* Value	CT Cohort	CR Cohort	*P* Value
Typical CIDP (n=37)	**Number**	n=23	n=14		n=23	n=14		n=23	n=14		n=23	n=14		n=23	n=14	
	**I-RODS**	57(18)	45(9.3)	***.01***	60(18)	63(12)	.12	58(21)	71(16)	***.005***	55(23)	85(15)	***.001***	58(37)	82(18)	***.002***
	**mRS**	3(2)	4(0.3)	***<.001***	3(1)	2(1)	.26	3(1)	1.5(1.3)	***.01***	3(2)	1(1)	***.001***	3(2)	1(1)	***.004***
	**INCAT**	4(2)	5(3.3)	***.04***	4(2)	3(1.5)	***.02***	3(1)	2(2)	***.004***	4(2)	1(1)	***<.001***	3(4)	1(1)	***<.001***
	**ONLS**	5(2)	6(3.3)	***.03***	5(2)	4(1.5)	***.05***	4(2)	2.5(2)	***.003***	5(2)	1(1)	***<.001***	4(4)	1.5(1.3)	***<.001***
	**TSS**	6.32(4.7)	8.65(2.7)	***.01***	6.32(4.9)	4.82(3.7)	.73	5.32(4.9)	3.82(4.7)	.26	5.66(5.7)	1.66(2.9)	***.05***	5.65(6.7)	1.33(4.2)	***.03***
	**COMPASS-31**	8(12)	14.5(17)	.08	10(10)	10.5(12)	.91	15(10)	7.5(5.3)	***.03***	13(11)	4.5(5.3)	***.001***	13(11)	4(6.25)	***.001***
CIDP Variants (n=36)	**Number**	n=17	n=19		n=17	n=19		n=17	n=19		n=17	n=19			n=17	n=19
	**I-RODS**	52(22)	61(15)	***.05***	57(23)	69(20)	***.007***	55(25)	88(17)	***<.001***	55(31.5)	88(20)	***<.001***	55(36)	88(15)	***<.001***
	**mRS**	3(1.5)	3(1)	.29	3(1.5)	2(2)	.06	3(4)	1(1)	***<.001***	3(2)	1(1)	***<.001***	3(1.5)	1(1)	***<.001***
	**INCAT**	4(2)	3(2)	.18	3(2)	2(2)	***.004***	4(2.5)	1(1)	***<.001***	3(4)	1(2)	***<.001***	3(4)	1(1)	***<.001***
	**ONLS**	5(2.5)	4(2)	.18	4(2.5)	3(2)	***.004***	5(3)	1(2)	***<.001***	4(4)	1(3)	***<.001***	4(4.5)	1(1)	***<.001***
	**TSS**	5.32(3.7)	7.32(3.9)	***.02***	5.32(3)	5.32(4.7)	.56	4.66(3.2)	2.66(2.7)	***.02***	4(3.3)	1.33(1.9)	***.001***	3.32(4.5)	1.33(1.9)	***.05***
	**COMPASS-31**	10(8.5)	7(12)	.56	10(10.5)	4(15)	.20	10(16)	3(9)	***.008***	9(16)	3(9)	***.03***	10(13)	3(6)	***.003***

Abbreviations: CT Cohort: Conventional Therapy Cohort; CR Cohort: Combined Rituximab Cohort; I-RODS: Inflammatory Rasch-Overall Disability Scale; mRS: modified Rankin Scale; INCAT: Inflammatory Neuropathy Cause and Treatment disability scale; ONLS: Overall Neuropathy Limitation Scale; TSS: Total Symptom Score; COMPASS-31: Composite Autonomic Symptom Score-31.

Supplemental Table 10. Transversal inter-subgroup analyses between CIDP subtypes in combined rituximab cohort.

Evaluation scales, median (IQR)	Baseline			1st visit			2nd visit			3rd visit			4th visit		
	Typical CIDP (n=14)	CIDP Variants (n=19)	*P* Value	Typical CIDP (n=14)	CIDP Variants (n=19)	*P* Value	Typical CIDP (n=14)	CIDP Variants (n=19)	*P* Value	Typical CIDP (n=14)	CIDP Variants (n=19)	*P* Value	Typical CIDP (n=14)	CIDP Variants (n=19)	*P* Value
**I-RODS**	45(9.25)	61(15)	***.001***	63(12)	69(20)	.21	71(16)	88(17)	.06	85(15)	88(20)	.38	82(17)	88(15)	.45
**mRS**	4(0.3)	3(1)	***<.001***	2(1)	2(2)	.38	1.5(1.3)	1(1)	.27	1(1)	1(1)	.91	1(1)	1(1)	.76
**INCAT**	5(3.3)	3(2)	***.01***	3(1.5)	2(2)	.45	2(2)	1(1)	.14	1(1)	1(2)	.59	1(1)	1(1)	.78
**ONLS**	6(3.2)	4(2)	***.005***	4(1.5)	3(2)	.15	2.5(2)	1(2)	.05	1(1)	1(3)	.47	1.5(1.3)	1(1)	.51
**TSS**	8.65(2.7)	7.32(3.9)	.29	4.82(3.7)	5.32(4.7)	.64	3.82(4.7)	2.66(2.7)	.26	1.66(2.9)	1.33(1.9)	.58	1.33(4.2)	1.33(1.9)	.74
**COMPASS-31**	14.5(17)	7(12)	.06	10.5(12.5)	4(15)	.05	7.5(5.3)	3(9)	.06	4.5(5.3)	3(9)	.73	4(6.3)	3(6)	.53

Abbreviations: I-RODS: Inflammatory Rasch-Overall Disability Scale; mRS: modified Rankin Scale; INCAT: Inflammatory Neuropathy Cause and Treatment disability scale; ONLS: Overall Neuropathy Limitation Scale; TSS: Total Symptom Score; COMPASS-31, Composite Autonomic Symptom Score-31.

Supplemental Table 11. Longitudinal intra-subgroup analyses of different CIDP subtype in combined rituximab cohort.

Subtype	Evaluation scales	Baseline Median (IQR)	1st visit Median (IQR)	*P* Value[a]	2nd visit Median (IQR)	*P* Value[a]	3rd visit Median (IQR)	*P* Value[a]	4th visit Median (IQR)	*P* Value[a]
Typical CIDP (n=14)	**I-RODS**	45(9.25)	63(12)	***<.001***	71(16)	***<.001***	85(15)	***<.001***	82(17)	***<.001***
	**mRS**	4(0.3)	2(1)	***<.001***	1.5(1.3)	***<.001***	1(1)	***<.001***	1(1)	***<.001***
	**INCAT**	5(3.3)	3(1.5)	***<.001***	2(2)	***<.001***	1(1)	***<.001***	1(1)	***<.001***
	**ONLS**	6(3.2)	4(1.5)	***<.001***	2.5(2)	***<.001***	1(1)	***<.001***	1.5(1.3)	***<.001***
	**TSS**	8.65(2.7)	4.82(3.7)	***.01***	3.82(4.7)	***<.001***	1.66(2.9)	***<.001***	1.33(4.2)	***<.001***
	**COMPASS-31**	14.5(17)	10.5(12.5)	.34	7.5(5.3)	***.01***	4.5(5.3)	***<.001***	4(6.3)	***<.001***
CIDP variants (n=19)	**I-RODS**	61(15)	69(20)	***.05***	88(17)	***<.001***	88(20)	***<.001***	88(15)	***<.001***
	**mRS**	3(1)	2(2)	.11	1(1)	***<.001***	1(1)	***<.001***	1(1)	***<.001***
	**INCAT**	3(2)	2(2)	.07	1(1)	***<.001***	1(2)	***<.001***	1(1)	***<.001***
	**ONLS**	4(2)	3(2)	***.02***	1(2)	***<.001***	1(3)	***<.001***	1(1)	***<.001***
	**TSS**	7.32(3.9)	5.32(4.7)	***.03***	2.66(2.7)	***<.001***	1.33(1.9)	***<.001***	1.33(1.9)	***<.001***
	**COMPASS-31**	7(12)	4(15)	.83	3(9)	.12	3(9)	.11	3(6)	***.02***

Abbreviations: I-RODS: Inflammatory Rasch-Overall Disability Scale; mRS: modified Rankin Scale; INCAT: Inflammatory Neuropathy Cause and Treatment disability scale; ONLS, Overall Neuropathy Limitation Scale; TSS, Total Symptom Score; COMPASS-31, Composite Autonomic Symptom Score-31.

[a] Comparison between baseline and each visit.

Supplemental Table 12. Transversal inter-subgroup analyses between early and delayed initiation of regimen in combined rituximab cohort.

Evaluation scales, median (IQR)	Baseline			1st visit			2nd visit			3rd visit			4th visit		
	Early initiation (< 10w, n=16)	Delayed initiation (≥ 10w, n=17)	*P* Value	Early initiation (< 10w, n=16)	Delayed initiation (≥ 10w, n=17)	*P* Value	Early initiation (< 10w, n=16)	Delayed initiation (≥ 10w, n=17)	*P* Value	Early initiation (< 10w, n=16)	Delayed initiation (≥ 10w, n=17)	*P* Value	Early initiation (< 10w, n=16)	Delayed initiation (≥ 10w, n=17)	*P* Value
**I-RODS**	56(19.3)	47(17.5)	.07	70(24)	63(10)	***.02***	88(17)	71(13)	***.03***	88(3.7)	76(18)	***.01***	84(14.3)	88(17)	.81
**mRS**	3(2)	4(1)	.09	2(1)	3(1)	***.02***	1(2)	2(1)	***.01***	1(0)	2(1)	***.03***	1(1)	1(1)	.75
**INCAT**	3(1.8)	5(2.5)	.16	2(2)	3(1)	.12	1(1)	2(2)	***.01***	1(0.8)	2(1)	***.03***	1(1)	1(1)	.71
**ONLS**	4(1)	6(2.5)	.23	2(2.8)	4(1)	.08	1(1.8)	2 (2)	***.02***	1(0.8)	2(2)	***.01***	1(1)	1(1.5)	.51
**TSS**	7.82(3.7)	7.32(2.8)	.55	5.32(4.3)	5.32(3.3)	.68	2.49(1.3)	4.32(3.7)	***.03***	1(1.8)	2.33(3.2)	***.01***	1.15(6.8)	1.33(1.7)	.86
**COMPASS-31**	7(12)	13(18)	.17	4(9.3)	11(10.5)	***.04***	3(6.5)	8(5)	***.05***	2(5.5)	6(7)	.06	2.5(5.5)	4(5)	.38

Abbreviations: I-RODS: Inflammatory Rasch-Overall Disability Scale; mRS: modified Rankin Scale; INCAT: Inflammatory Neuropathy Cause and Treatment disability scale; ONLS: Overall Neuropathy Limitation Scale; TSS: Total Symptom Score; COMPASS-31, Composite Autonomic Symptom Score-31.

Supplemental Table 13. Longitudinal intra-subgroup analyses for different initiation opportunity in combined rituximab cohort.

Opportunity	Evaluation scales	Baseline Median (IQR)	1st visit Median (IQR)	*P* Value[a]	2nd visit Median (IQR)	*P* Value[a]	3rd visit Median (IQR)	*P* Value[a]	4th visit Median (IQR)	*P* Value[a]
Early initiation (<10 weeks, n=16)	I-RODS	56(19.3)	70(24)	***.015***	88(17)	***<.001***	88(3.7)	***<.001***	84(14.3)	***<.001***
	mRS	3(2)	2(1)	***.03***	1(2)	***<.001***	1(0)	***<.001***	1(1)	***<.001***
	INCAT	3(1.8)	2(2)	***.05***	1(1)	***<.001***	1(0.8)	***<.001***	1(1)	***<.001***
	ONLS	4(1)	2(2.8)	***.006***	1(1.8)	***<.001***	1(0.8)	***<.001***	1(1)	***<.001***
	TSS	7.82(3.7)	5.32(4.3)	***.02***	2.49(1.3)	***<.001***	1(1.8)	***<.001***	1.15(6.8)	***<.001***
	COMPASS-31	7(12)	4(9.3)	.44	3(6.5)	.12	2(5.5)	***.04***	2.5(5.5)	***.03***
Delayed initiation (≥10 weeks, n=17)	I-RODS	47(17.5)	63(10)	***.006***	71(13)	***<.001***	76(18)	***<.001***	88(17)	***<.001***
	mRS	4(1)	3(1)	***.004***	2(1)	***<.001***	2(1)	***<.001***	1(1)	***<.001***
	INCAT	5(2.5)	3(1)	***.001***	2(2)	***<.001***	2(1)	***<.001***	1(1)	***<.001***
	ONLS	6(2.5)	4(1)	***.001***	2 (2)	***<.001***	2(2)	***<.001***	1(1.5)	***<.001***
	TSS	7.32(2.8)	5.32(3.3)	*.11*	4.32(3.7)	***.003***	2.33(3.2)	***<.001***	1.33(1.7)	***<.001***
	COMPASS-31	13(18)	11(10.5)	.35	8(5)	***.01***	6(7)	***.001***	4(5)	***<.001***

Abbreviations: I-RODS, Inflammatory Rasch-Overall Disability Scale; mRS, modified Rankin Scale; INCAT, Inflammatory Neuropathy Cause and Treatment disability scale; ONLS, Overall Neuropathy Limitation Scale; TSS, Total Symptom Score; COMPASS-31, Composite Autonomic Symptom Score-31.

[a] Comparison between baseline and each visit.
