# Supplementary material for: Efficacy and safety of combined low‐dose rituximab regimen for chronic inflammatory demyelinating polyradiculoneuropathy
Source: Ann Clin Transl Neurol. 2024 Dec 11;12(1):180–91. doi: 10.1002/acn3.52270 (PMC11752089; doi:10.1002/acn3.52270)
Supplement: Supplementary file 3 — Captions. [file ACN3-12-180-s002.docx]

**Figure S1.** **Comparisons of clinical improvements between conventional therapy and combined rituximab cohort in CIDP.** During the follow-up, combined rituximab cohort showed better improvements in mRS (A), INCAT (B), ONLS (C), TSS (D), COMPASS 31 scores (E) at each visit than those in conventional therapy cohort. *p < 0.05, **p < 0.01, ***p < 0.001

**Figure S2.** **Comparisons of proportions of favorable response between conventional therapy and combined rituximab cohort.** During the follow-up, combined rituximab cohort showed higher proportions of favorable response in mRS (A), INCAT (B), ONLS (C), TSS (D), COMPASS 31 scores (E) at each visit than those in conventional therapy cohort. ***p < 0.001

**Figure S3.** **Comparisons of deterioration occurrence between conventional therapy and combined rituximab cohort.**

During the follow-up of 52 weeks, combined rituximab cohort showed higher non-deterioration probability in mRS (A), INCAT (B), ONLS (C) and TSS (D) than those in conventional therapy cohort.

**Figure S4.** **Longitudinal intra-cohort analyses of favorable outcome in conventional therapy and combined rituximab cohort.** During the follow-up, proportions of favorable outcome in mRS (A), INCAT (C), ONLS (E) and TSS (G) were not different at each visit in conventional therapy cohort, while the proportions were significantly increased in mRS (B), INCAT (D), ONLS (F) and TSS (H) during the first three visits within 28 weeks in combined rituximab cohort, and stably sustained till the last visit of 52 weeks. *p < 0.05, **p < 0.01

**Figure S5.** **Comparisons of clinical improvements between typical CIDP and CIDP variants subgroup in combined rituximab cohort.** During the follow-up, the favorable improvements of mRS (A), INCAT (B), ONLS (C), TSS (D), COMPASS 31 scores (E) were not different at each visit between the typical CIDP and CIDP variants subgroup in combined rituximab cohort. *p < 0.05, **p < 0.01, ***p < 0.001

**Figure S6.** **Comparisons of clinical improvements between early and delayed initiation of combined rituximab regimen subgroup in combined rituximab cohort.** During the follow-up, early initiating combined rituximab regimen (<10 weeks) showed better improvements in mRS (A), INCAT (B), ONLS (C), TSS (D), COMPASS 31 scores (E) than delayed initiation subgroup (≥10 weeks) at the first three visits within 28 weeks, while had no differences in favorable prognoses at the last visit of 52 weeks. *p < 0.05
